# Molecular Nanomachines Can Destroy Tissue or Kill Multicellular Eukaryotes

**DOI:** 10.1021/acsami.9b22595

**Published:** 2020-03-13

**Authors:** Richard S. Gunasekera, Thushara Galbadage, Ciceron Ayala-Orozco, Dongdong Liu, Victor García-López, Brian E. Troutman, Josiah J. Tour, Robert Pal, Sunil Krishnan, Jeffrey D. Cirillo, James M. Tour

**Affiliations:** Department of Chemistry, Rice University, Houston, Texas 77005, United States; Department Biological Sciences and Department of Chemistry, Physics and Engineering, Biola University, La Mirada, California 90639, United States; Department of Microbial Pathogenesis and Immunology, Texas A&M Health Science Center, Bryan, Texas 77807, United States; Department of Chemistry, Rice University, Houston, Texas 77005, United States; Department of Experimental Oncology, MD Anderson Cancer Center, Houston, Texas 77030, United States; Department of Chemistry, Rice University, Houston, Texas 77005, United States; Department of Physics and Astronomy, Rice University, Houston, Texas 77005, United States; Department of Microbial Pathogenesis and Immunology, Texas A&M Health Science Center, Bryan, Texas 77807, United States; Department of Chemistry, Durham University, DH1 3LE Durham, United Kingdom; Department of Experimental Oncology, MD Anderson Cancer Center, Houston, Texas 77030, United States; Department of Microbial Pathogenesis and Immunology, Texas A&M Health Science Center, Bryan, Texas 77807, United States; Department of Chemistry and Department of Materials Science and NanoEngineering, Smalley-Curl Institute, and NanoCarbon Center, Rice University, Houston, Texas 77005, United States

**Keywords:** molecular nanomachines, cancer cells, bacteria, C. elegans, parasite, Daphnia, mouse, in vivo

## Abstract

Light-activated molecular nanomachines (MNMs) can be used to drill holes into prokaryotic (bacterial) cell walls and the membrane of eukaryotic cells, including mammalian cancer cells, by their fast rotational movement, leading to cell death. We examined how these MNMs function in multicellular organisms and investigated their use for treatment and eradication of specific diseases by causing damage to certain tissues and small organisms. Three model eukaryotic species, *Caenorhabditis elegans*, *Daphnia pulex*, and *Mus musculus* (mouse), were evaluated. These organisms were exposed to light-activated fast-rotating MNMs and their physiological and pathological changes were studied in detail. Slow rotating MNMs were used to control for the effects of rotation rate. We demonstrate that fast-rotating MNMs caused depigmentation and 70% mortality in *C. elegans* while reducing the movement as well as heart rate and causing tissue damage in *Daphnia*. Topically applied light-activated MNMs on mouse skin caused ulceration and microlesions in the epithelial tissue, allowing MNMs to localize into deeper epidermal tissue. Overall, this study shows that the nanomechanical action of light-activated MNMs is effective against multicellular organisms, disrupting cell membranes and damaging tissue *in vivo*. Customized MNMs that target specific tissues for therapy combined with spatial and temporal control could have broad clinical applications in a variety of benign and malignant disease states including treatment of cancer, parasites, bacteria, and diseased tissues.

## Graphical Abstract

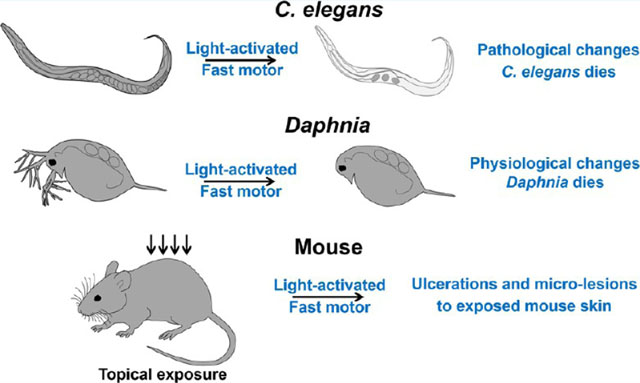

## INTRODUCTION

Molecular nanomachines (MNMs) are synthetically engineered molecules that have light-induced actuation (motorization). The design of these nanomachines was conceptually proposed in 1961,^1^ but the recent award of the 2016 Nobel Prize for Chemistry for their discovery emphasizes the current high level of interest in their potential applications.^2^ To better understand potential applications for MNM, it is important to evaluate how they can be applied in different cells and tissues. Of particular interest here is their functioning *in vivo* in multicellular organisms.

*Caenorhabditis elegans* is a hermaphrodite nematode worm, a free-living organism found on moist soil, and is a leading model organism used widely in neurobiology, biomedicine, genetics, and the study of host–pathogen interactions.^3–6^ *C. elegans* has a life span of 15–20 days at ambient temperatures from 20 to 22 °C.^7,8^ It was first used as a model organism to study the mechanisms of aging.^9^ Moreover, *C. elegans* has a transparent body that permits the visualization of fluorescently tagged molecules, pathogens, and tissue pathologies.^6^ Molecular work in *C. elegans* over the past two decades has led to the discovery of important molecular mechanisms including programmed cell death,^10^ RNAi techniques for gene silencing,^11^ and expression of the green fluorescent protein (GPF).^12^ These molecular characteristics make *C. elegans* a desirable model to study physiological and pathological changes caused by external agents or treatments.

*Daphnia* species, commonly known as water fleas, are crustacean filter feeders in freshwater reservoirs. *Daphnia* is a commonly used model organism in ecological studies examining different toxicities and environmental factors affecting freshwater systems.^13,14^ More recently, *Daphnia pulex* and *Daphnia magna* have been used to study the effects of nanoparticles and their impact on the environment.^15^ *Daphnia* offers several advantages as a model system including ease of handling and visible characteristics for experimental measurements. Small invertebrate models such as *C. elegans* and *Daphnia* allow light to penetrate the organism and make it possible to study morphological changes.

Hairless nude mice are used to study skin pathology, lesions, and ulcerations associated with skin cancers, including melanoma and adenocarcinoma.^16–19^ These mouse skin models are used to observe skin histopathology together with fluorescent microscopy to identify localized cellular processes.^20^

We previously showed that MNMs can be used to open cellular membranes and synthetic lipid bilayers, destroy cancer cells, and disrupt bacterial cell wells *in vitro*.^21,22^ We sought to determine whether these MNMs can work effectively *in vivo*, retaining their disruptive nature in a spatially controlled manner when introduced to whole organisms. We used *C. elegans*, *Daphnia*, and hairless nude mice to study the morphological effects of light-activated fast motor MNMs in small eukaryotes and mammalian tissue. We studied different destructive effects that MNMs cause in these eukaryotes *in vivo* using several metrics including physiological and histopathological changes and mortality rates. Our *in vivo* study expands the application of MNMs from their nanomechanical action on *in vitro* cell lines to whole organisms ranging from small microscopic invertebrates to larger mammalian vertebrates. Our results show that MNMs cause damage to cells and tissues in these eukaryotic species to varying degrees.

## RESULTS AND DISCUSSION

**Figure 1.**
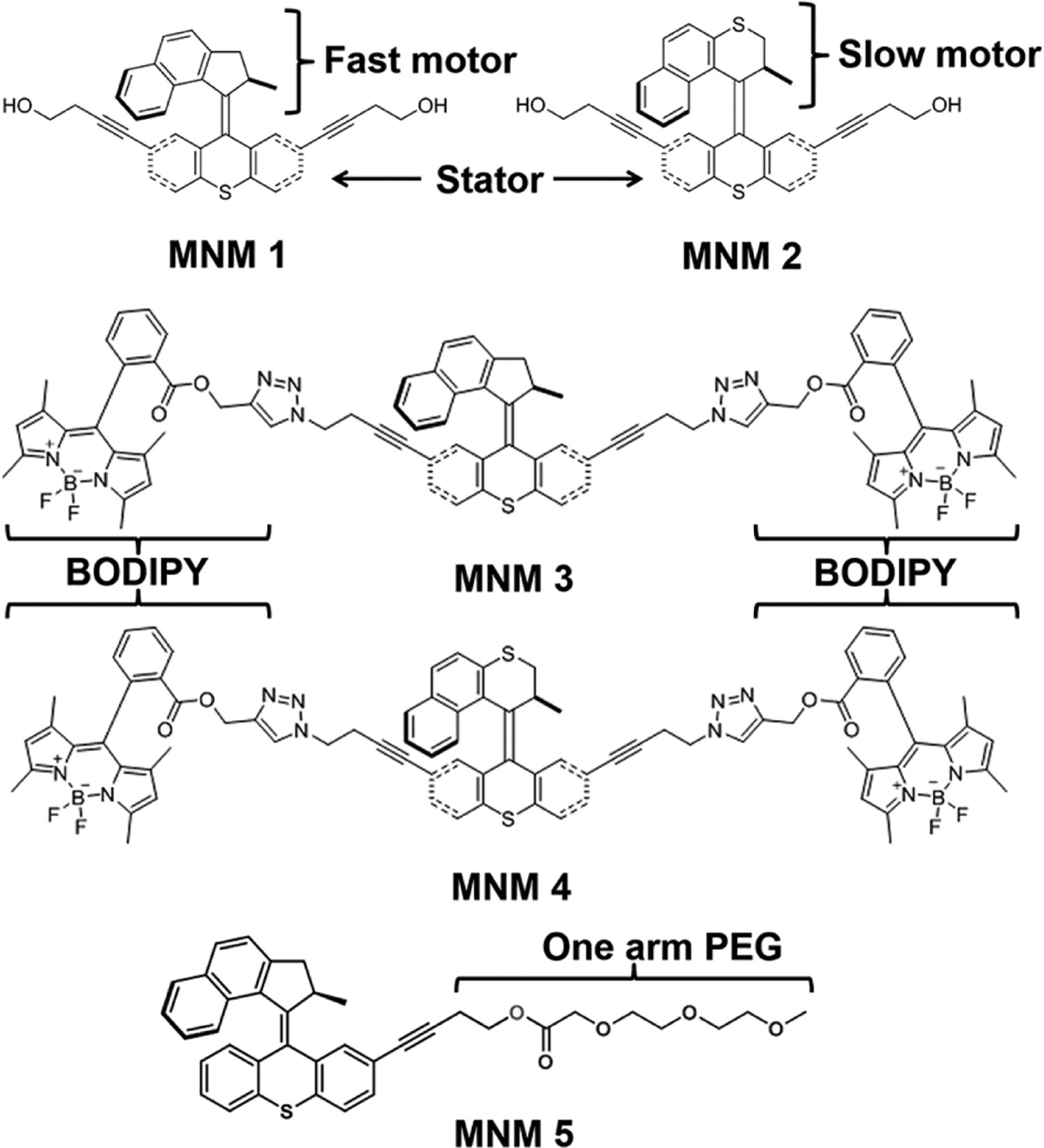
MNMs used in this study. MNM **1**: a fast motor with a rotor that can rotate at 2–3 MHz when activated with 365 nm light. MNM **2**: a slow motor with a rotor that can rotate 1.8 revolutions h^−1^ when activated with 365 nm light at 60 °C but only undergoes cis/trans isomerization at lower temperatures, not a full rotation. MNM **3**: a fast motor with BODIPY fluorophores attached to two arms in its stator. MNM **4**: a slow motor with BODIPY fluorophore attached to two arms in its stator. MNM **5**: a fast motor with one PEG to increase water solubility.

When MNMs (Figure 1) were added in micromolar concentrations (1–10 *μ*M) into mammalian cells cultured in media, and activated by 365 nm light, they embeded into the cell membrane and made pores by using their fast-rotational movement. The rotational speed of MNM **1** is 2–3 million rotations s^−1^. MNMs that bear peptides for specific cell targeting can be activated at 355–365 nm to destroy prostate adenocarcinoma cells (PC-3) and mouse embryonic fibroblast cells (NIH 3T3) *in vitro*.^21^ The variations of MNMs used in this study are shown in Figure 1. The slow motor (MNM **2**) rotates at ~1.8 revolutions h^−1^ and allows us to control for the effects of MNMs other than fast rotational movement. We attached BODIPY fluorophores to the stator of MNM **1** to form MNM **3**, and we use ^1^H NMR spectra to characterize the half-rotation of slow MNM **4** (Figures S1 and S2). Hence, MNM **2** and MNM **4** serve as controls to confirm that the deleterious effects observed with MNM **1** and MNM **3** are due to their drilling action via fast rotational movements. MNM **5** provides a complementary water-soluble pendant to the fast motor core of MNM **1**.

### Light-Activated MNM 1 Can Cause Pathological Changes in *C. elegans*.

**Figure 2.**
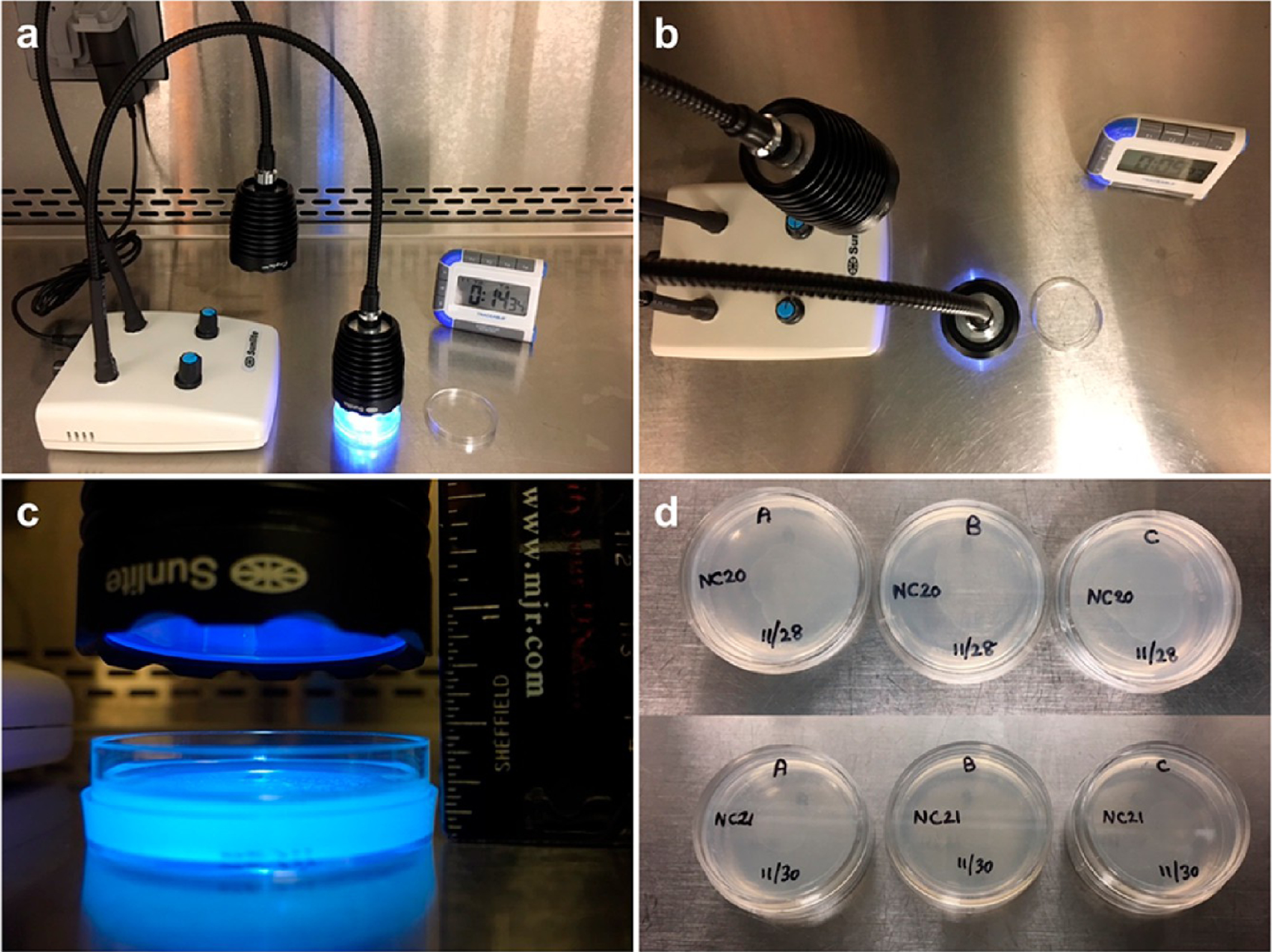
*C. elegans* experimental setup. Nematodes incubated with MNM for 15 min were transferred onto NGM agar plates and exposed to 365 nm light. (a) Light source with a wavelength of 365 nm. (b) The light source was placed directly above the NGM agar plate to cover the entire plate and ensure high and constant energy delivery. (c) *C. elegans* in NGM agar plate with 365 nm light source at a constant distance of 1.27 cm above the agar plate to deliver a constant energy of 10–15 mW/cm^2^. (d) NMG agar plates with *E. coli* (OP50) as a food source used to maintain *C. elegans* after MNM and light exposure.

**Figure 3.**
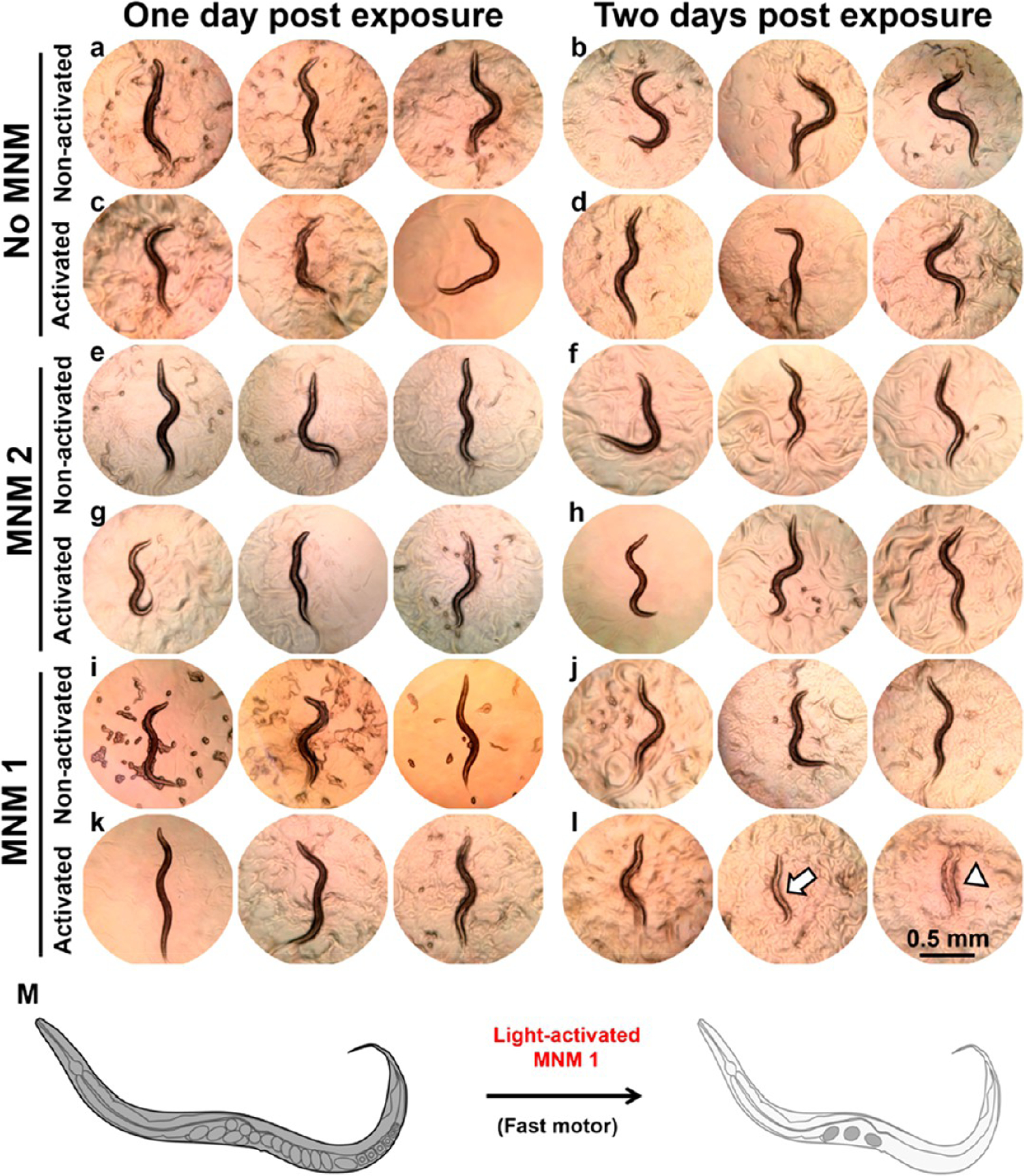
Light-activated MNM **1** can cause pathological changes in *C. elegans*. *C. elegans* exposed to either nonactivated or 365 nm light-activated MNM **1**, MNM **2**, or no MNM observed under a light microscope (40× 1.3 NA) to identify morphological and pathological changes. *C. elegans* in all groups were incubated with MNM in 10% DMSO for 15 min prior to light activation. Three representative *C. elegans* are shown for each group; 1 day and 2 days postexposure to MNM. (a, b) Incubated with only 10% DMSO (no MNM), without light activation. (c, d) Incubated with no MNM, with light activation. (e, f) Incubated with MNM **2**, without 365 nm light activation. (g, h) Incubated with MNM **2**, with light activation. (i, j) Incubated with MNM **1**, without light activation. (k, l) Incubated with MNM **1**, with light activation. (l) White arrow shows *C. elegans* with loss of pigmentation, and the white arrowhead shows two dead *C. elegans*, 2 days postactivation. The scale bar in the lower right is the same for all images. (m) Schematic of a healthy 3 day old *C. elegans* exposed to light-activated MNM **1** showing depigmentation, shortened length, and reduced progeny, eventually leading to its death.

We used a 365 nm light source to activate MNMs with constant light flux (10–15 mW/cm^2^) delivered at a constant distance from our model organisms (Figure 2). To study morphological and pathological effects caused by the fast rotational action of MNM **1**, we exposed N2 *C. elegans* (wild type) in 10% DMSO with no MNMs, MNM **2** (100 *μ*M), or MNM **1** (100 *μ*M) with and without light activation for 15 min. DMSO was needed at 1% or 10% to dissolve MNMs according to their concentrations. *C. elegans* were then observed under a light microscope (40× magnification) to identify changes caused by MNM (Figure 3). Control groups of *C. elegans* were exposed to no MNM or MNM **2** (100 *μ*M) with and without light activation, resulting in healthy nematodes with appropriate dark pigmentation and no apparent pathological changes (Figure 3a–h). In addition, it was observed that their ability to lay eggs was intact, 1 day and 2 days postexposure. In contrast, we observed *C. elegans* exposed to light-activated MNM **1** (100 *μ*M) showed a notable decrease in the number of eggs laid (>70% reduction in eggs laid compared to nonexposed *C. elegans*) and a decrease in pigmentation when compared to non-light-activated MNM **1** exposed *C. elegans* (Figure 3i–l) at 1 day and 2 days postexposure. Our observations suggest that light-activated MNM **1** can cause deleterious effects in *C. elegans* as early as 1 day postexposure.

*C. elegans* in control groups that were exposed to either no MNM (Figure 3b,d) or slow MNM **2** (100 *μ*M) control (Figure 3f, h), 2 days postexposure, retained their dark pigmentation and body length and survived the initial exposure. In contrast, those exposed to light-activated MNM **1** (100 *μ*M) (Figure 3l) showed pathological changes 2 days postexposure. This included loss of dark pigmentation (arrow), a decrease in length (arrow), and eventually leading to death (arrowhead). This was a gradual process over the first 2 days postexposure, with loss of dark pigmentation observed even 1 day after exposure (Figure 3k). Our results indicate that MNM **1** (100 *μ*M) can cause pathological changes in *C. elegans* with as little as 15 min of light activation. *C. elegans* exposed to light-activated MNM **1** underwent depigmentation, displayed a shortened length, had a >70% reduction in laying of eggs, and resulted in the death of *C. elegans* (Figure 3m).

### Light-Activated MNM 1 Causes Increased Mortality in *C. elegans*.

**Figure 4.**
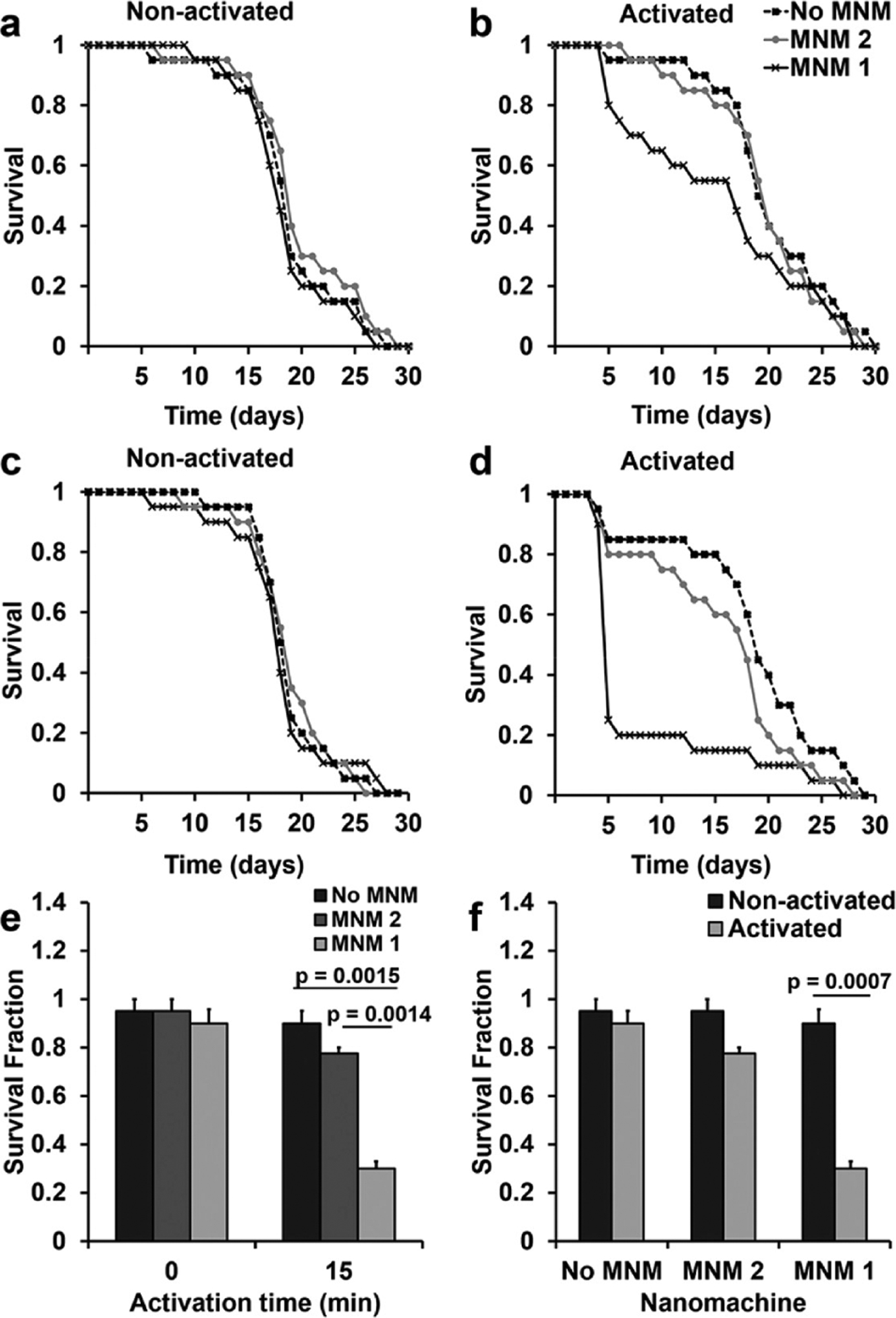
Light-activated MNM **1** increases mortality in *C. elegans*. Three-day-old adult *C. elegans* incubated with 100 *μ*M MNM **1**, MNM **2**, or no MNM control for 15 min and exposed to 365 nm light for 15 min. The life span and mortality rate were assayed by counting *C. elegans* daily. Each group had three replicates of 20 nematodes each (total 60 per group). (a, b) *C. elegans* incubated with no MNM, MNM **2**, and MNM **1**, in 1% DMSO. (a) Without light activation (*P* = 0.432). (b) With 15 min of 365 nm light activation (*P* = 0.309). (c, d) *C. elegans* incubated with no MNM, MNM **2**, and MNM **1**, in 10% DMSO. (c) Without light activation (*P* = 0.981). (d) With 15 min of light activation (*P* = 0.005). (e, f) Survival fraction of *C. elegans* incubated with MNM **1**, MNM **2**, or no MNM, in 10% DMSO, with or without 365 nm light activation, 2 days postexposure. *C. elegans* exposed to 15 min of light-activated MNM **1** had a 70% mortality rate. (e) Comparing differences in survival fraction between no MNM, MNM **2**, and MNM **1**. (f) Comparing differences in survival fraction between light-activated and non-light-activated in each of the MNMs. The bars represent the mean of each group, and the error bars represent the standard error. *P*-values in (a–d) calculated with log-rank Mantel–Cox tests and (e, f) with unpaired two-tail *t* tests and horizontal bars indicate the two groups compared.

To study the effects of MNM **1** on mortality, we incubated *C. elegans* without MNM, with 100 *μ*M MNM **2**, or with 100 *μ*M MNM **1** for 15 min in 1% or 10% DMSO and activated with 365 nm light for 15 min. Without light activation *C. elegans* exposed to no MNM, MNM **2** (100 *μ*M), or MNM **1** (100 *μ*M) in 1% DMSO displayed similar life spans (Figure 4a). With light activation, *C. elegans* exposed to MNM **1** (100 *μ*M) in 1% DMSO showed a mortality rate of 25% 2 days postexposure (Figure 4b). In groups exposed to light-activated MNM **1** (100 *μ*M) with 10% DMSO, we observed a mortality rate of 70% 2 days postexposure compared to groups not activated by light (Figure 4c–f). This was in contrast to mortality rates that were seen with MNM **2** (100 *μ*M) (22.5%) and no MNM control (10%) groups (Figure 4c–f). *C. elegans* exposed to 100 *μ*M MNM **1** or **2** without light activation showed no increase in mortality, similar to that of the no MNM control group (Figure 4a,c). These results suggest that MNM induced mortality is specifically driven by light activation of fast MNM **1**, and the effect is concentration-dependent. *C. elegans* exposed to 365 nm light for 15 min (Figure 4b,d) showed a slight increase in mean life span compared to those not exposed to 365 nm light (Figure 4a,c). Such an increase in life span has been observed before and is attributed to the induction of stress proteins when *C. elegans* is exposed to a stressor such as heat or light energy.^8,23^

**Figure 5.**
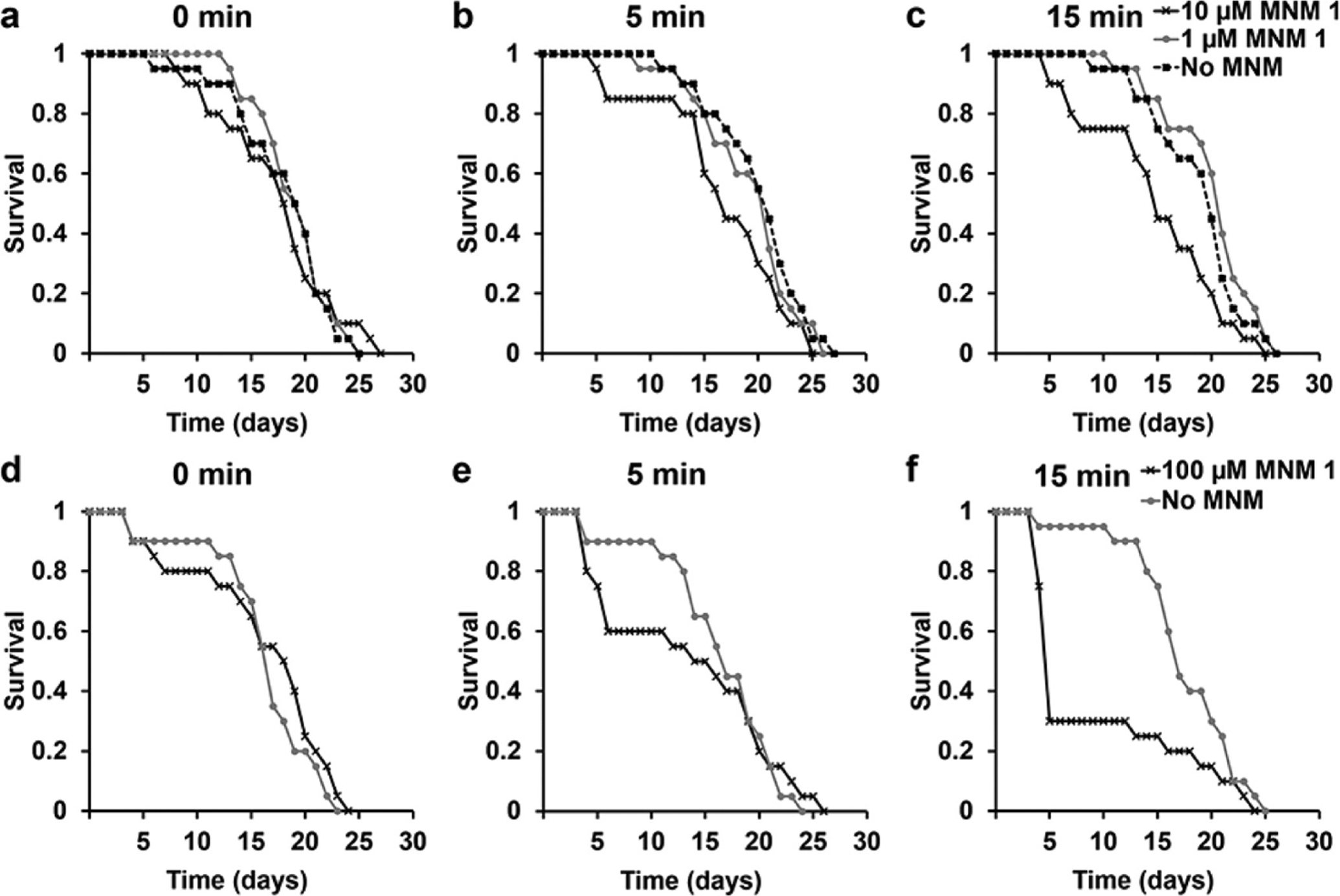
*C. elegans* exposed to 0, 1, and 10 *μ*M MNM **1** in 1% (a–c) or 10% DMSO (d–f). (a–c) Three-day-old adult *C. elegans* incubated with no MNM, 1 *μ*M, or 10 *μ*M MNM **1** in 1% DMSO for 15 min and activated with 365 nm light. The life span and mortality rate were assayed by counting *C. elegans* daily. Each group had 20 nematodes. (a) Without light activation (*P* = 0.987). (b) 5 min of light activation (*P* = 0.244). (c) 15 min of light activation (*P* = 0.016). (d–f) Three-day-old adult *C. elegans* incubated with no MNM or 100 *μ*M MNM **1** in 10% DMSO for 15 min and activated with 365 nm light. The life span and mortality rate were assayed by counting *C. elegans* daily. Each group had 20 nematodes. (d) Without light activation (*P* = 0.372). (e) 5 min of light activation (*P* = 0.887). (f) 15 min of light activation (*P* = 0.019). *P*-values in (a–f) were calculated with log-rank Mantel–Cox tests.

We examined the dose–response of light activation on mortality caused by MNM **1** (100 *μ*M) through exposing *C. elegans* to 5 min of light activation, 15 min of light activation, or no light activation. Activation of MNM **1** (100 *μ*M) with 365 nm light showed a time-dependent increase in mortality (Figure 5). The mortality of *C. elegans* was also dependent on the concentration of activated MNM **1** (Figure 5a–c). *C. elegans* exposed to MNM **1** at 100 *μ*M with 15 min of light activation showed a 65% mortality compared to 25% with 5 min of light activation and 10% mortality without light activation, 2 days postexposure (Figure 5e,f). These results further confirm that mortality caused by MNM **1** (100 *μ*M) is due to their fast rotational movement triggered by 365 nm light activation, and the concentration and time dependence suggests a dose–response relationship.

### Presence of DMSO Increases Light-Activated MNM 1 Mediated *C. elegans* Mortality.

We were interested in whether DMSO plays a role in the observed *C. elegans* mortality when exposed to MNM **1** (100 *μ*M). *C. elegans* exposed to light-activated MNM **1** (100 *μ*M) had higher mortality rates when incubated with 10% DMSO (70% mortality, Figures 4c,d and 5d–f) when compared to 1% DMSO (25% mortality, Figures 4a,b and 5a–c), 2 days postexposure. *C. elegans* exposed to light-activated MNM **1** (100 *μ*M) in 1% DMSO continued to die, showing a mortality rate of 45% over the next 5 days. This showed that even though DMSO alone did not have a deleterious effect on *C. elegans* when light-activated MNM **1** (100 *μ*M) were combined with 10% DMSO, they increased mortality from 25% to 70%. In the presence of 10% DMSO, the mortality rate of *C. elegans* treated with light-activated MNM **2** (100 *μ*M) also increased slightly to 20%. But this was not significantly different from the mortality caused by the no MNM, with a mortality rate of 15% (Figure 4d–f). In the absence of light activation, 10% DMSO with or without MNM did not cause an increase in mortality (Figure 4c,e). This suggests that DMSO, by itself, does not cause mortality. However, DMSO increases MNM **1** mediated mortality. This is likely due to the increase in cell permeability caused by DMSO that allows MNM **1** to gain access to cells and thereby cause more deleterious effects.

### Light-Activated MNM 3 Increases Gut Autofluorescence in *C. elegans*.

**Figure 6.**
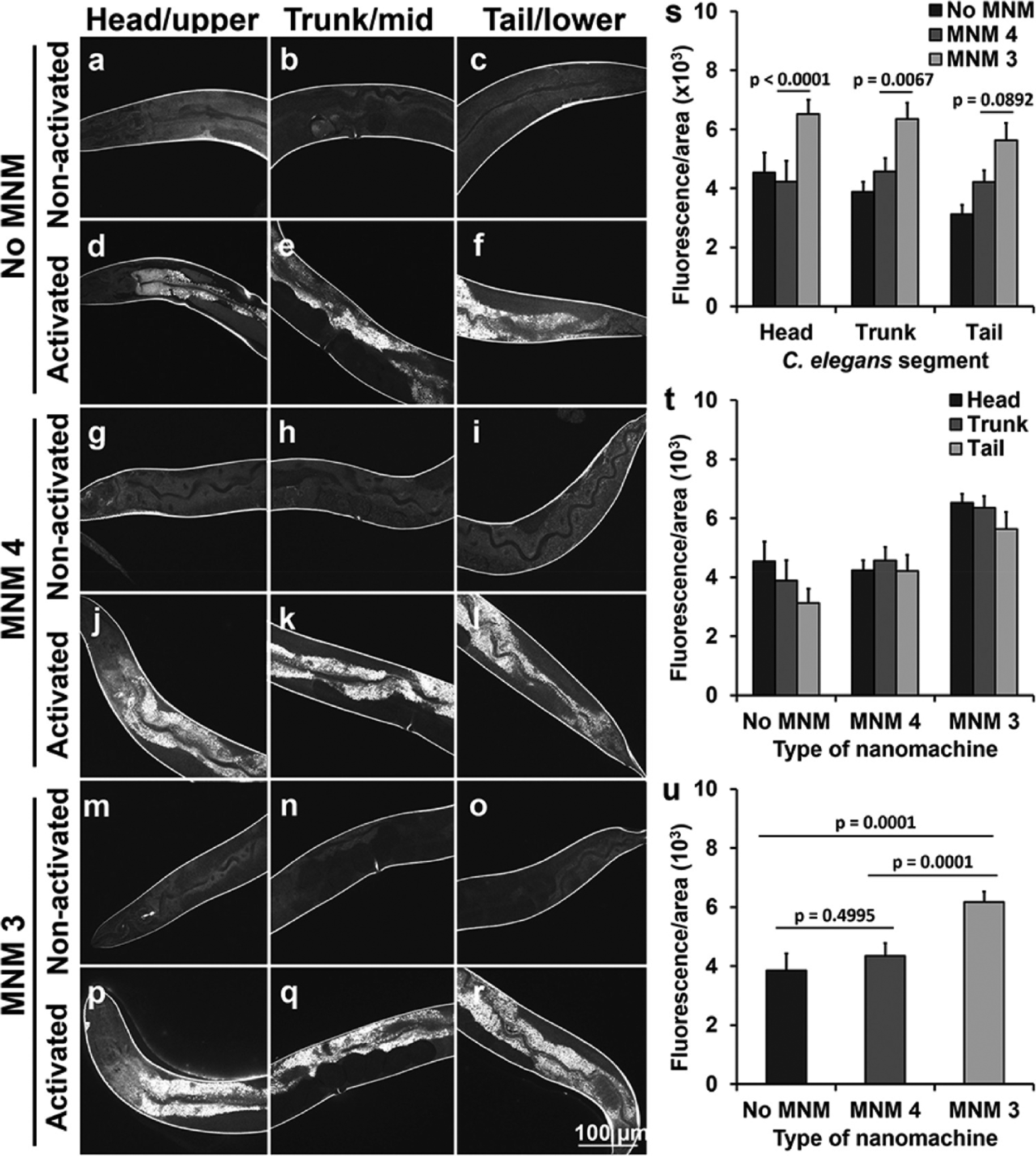
High-resolution confocal images show an increase in autofluorescence in *C. elegans* exposed to light-activated MNM **3**. TP12 *C. elegans* (expressing eGFP in the cuticle) exposed to 100 *μ*M MNM **4** or MNM **3** for 15 min in 10% DMSO were imaged with or without 365 nm light activation. A 40× 1.3 NA oil-immersion objective was used, with an excitation wavelength of 488 nm. At least four *C. elegans* were imaged for each group. Each nematode was imaged head (upper segment), trunk (midsegment), and tail (lower segment). Representative images are shown for each group. (a–f) *C. elegans* incubated with 10% DMSO (no MNM). (a–c) Without 365 nm light exposure. (d–f) With 15 min of light exposure. (g–l) *C. elegans* incubated with 100 *μ*M MNM **4** with 10% DMSO. (g–i) Without 365 nm light activation. (j–l) With 15 min of light activation. (m–r) *C. elegans* incubated with 100 *μ*M MNM **3** with 10% DMSO. (m–o) Without 365 nm light activation. (p–r) With 15 min of light activation. (s–u) Quantification of autofluorescence in *C. elegans* in each group. Four nematodes were used to evaluate three areas of fluorescence in each nematode, giving a mean fluorescence with 12 measurements for each group. The relative increase in fluorescence is shown after correcting for autofluorescence in *C. elegans* exposed to nonactivated MNM. (s) Relative increase in autofluorescence in no MNM, MNM **3**, and MNM **4** groups for each segment (upper, middle, and lower). *C. elegans* incubated with 365 nm light-activated MNM **3** showed a significant increase in autofluorescence. (t) Relative increase in autofluorescence for each of the segments (upper, middle, and lower) is similar in no MNM, MNM **3**, and MNM **4**. (u) Relative increase in autofluorescence for *C. elegans* as a whole organism. Light-activated MNM **3** showed a significant increase compared to both MNM **4** and no MNM groups. The bars represent the mean of each group, and the error bars represent the standard error. *P*-values are from unpaired two-tail *t* tests, and the horizontal bars indicate the two groups compared. The scale bar at the lower right is the same for all images.

We examined the physiological and pathological effects of MNM **1** treatment of *C. elegans*. We used a *C. elegans* strain expressing eGFP on its cuticle (TP12 strain) with MNM **3** and MNM **4** and examined them via confocal microscopy. While we did not observe widespread microlesions on the cuticular surface of *C. elegans* at this resolution, we observed a significant increase in gut autofluorescence in *C. elegans* exposed to 365 nm light-activated fast MNM **3** compared to *C. elegans* exposed to no MNM or slow MNM **4** (Figure 6). Gut autofluorescence, among other factors, is a marker of increased stress in *C. elegans*.^24^ While there was an observable increase in gut autofluorescence in all *C. elegans* subjected to 365 nm light, the increase in autofluorescence was more widespread and intense in those exposed to light-activated MNM **3** (Figure 6a–r). *C. elegans* incubated with either no MNM or MNM **4** displayed the same level of autofluorescence that was significantly lower than MNM **3** (Figure 6d–f,j–l,s–u). These results suggest that while 365 nm light alone can cause some stress in *C. elegans*, light-activated MNM **3** causes even greater stress (Figure 6p–r, s–u). The most likely source of stress in *C. elegans* is the fast rotational movement of the light-activated MNM **3** damaging tissues.

Autofluorescence in animal tissue is mainly due to intra-cellular lysosome-derived granules, extracellular collagen, or mitochondrial products (NADPH and flavins).^25,26^ Autofluorescent molecules lipofuscin and glycation end-products accumulate during the aging process in *C. elegans*.^25^ Gut autofluorescence is also a marker for increased stress and stress response in *C. elegans*.^24,27^ Our observations suggest that light-activated MNM **3** causes an increase in stress in *C. elegans*, increasing their autofluorescence. This is to be expected as the light-activated fast-rotating MNM causes mechanical damage to the surrounding tissues.

### Light-Activated MNM 1 Causes Physiological Changes in *Daphnia*.

**Figure 7.**
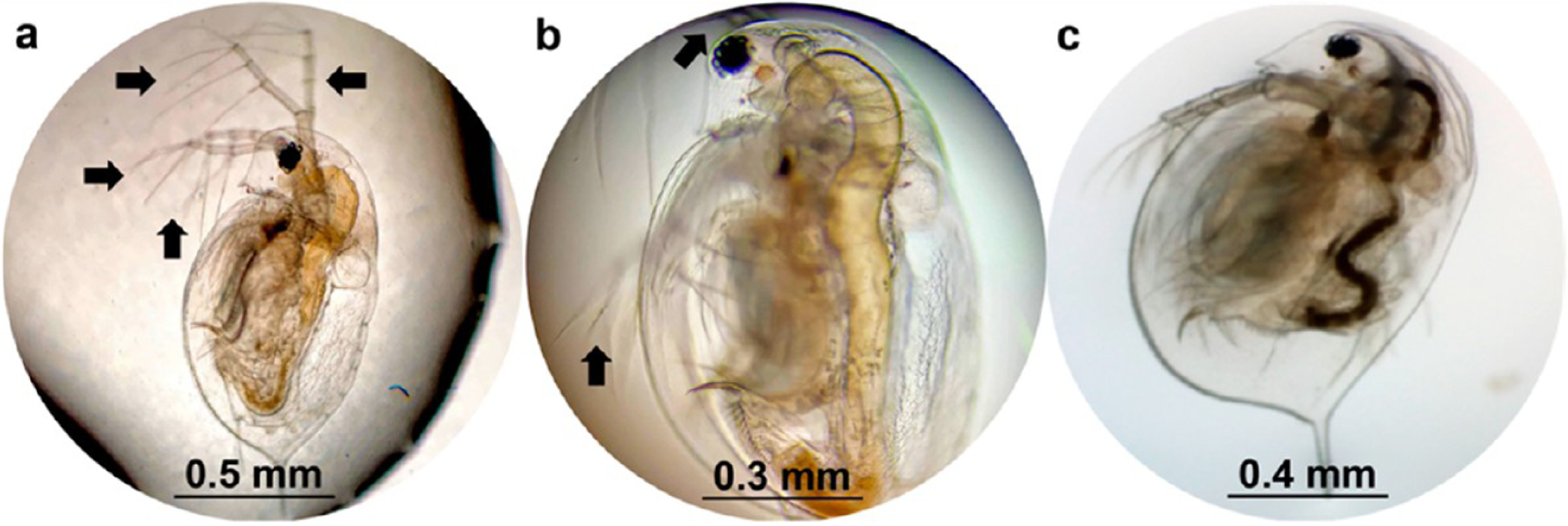
Representative *Daphnia* images from the experimental setup. (a–c) *Daphnia* in a water droplet incubated with 1 or 10 *μ*M MNM **1** for 10 min as observed under a light microscope with a 4× 0.13 NA air objective. (a) A healthy adult *Daphnia* with all four of its appendages (arrows), as observed under a light microscope with a 4× 0.13 NA air objective. (b) An adult *Daphnia* as observed under a light microscope after light-activated MNM **1** treatment, showing only two of its four appendages intact (arrows). (c) A deceased adult *Daphnia* as observed under a light microscope 5 days post-light-activated MNM **1** treatment.

**Figure 8.**
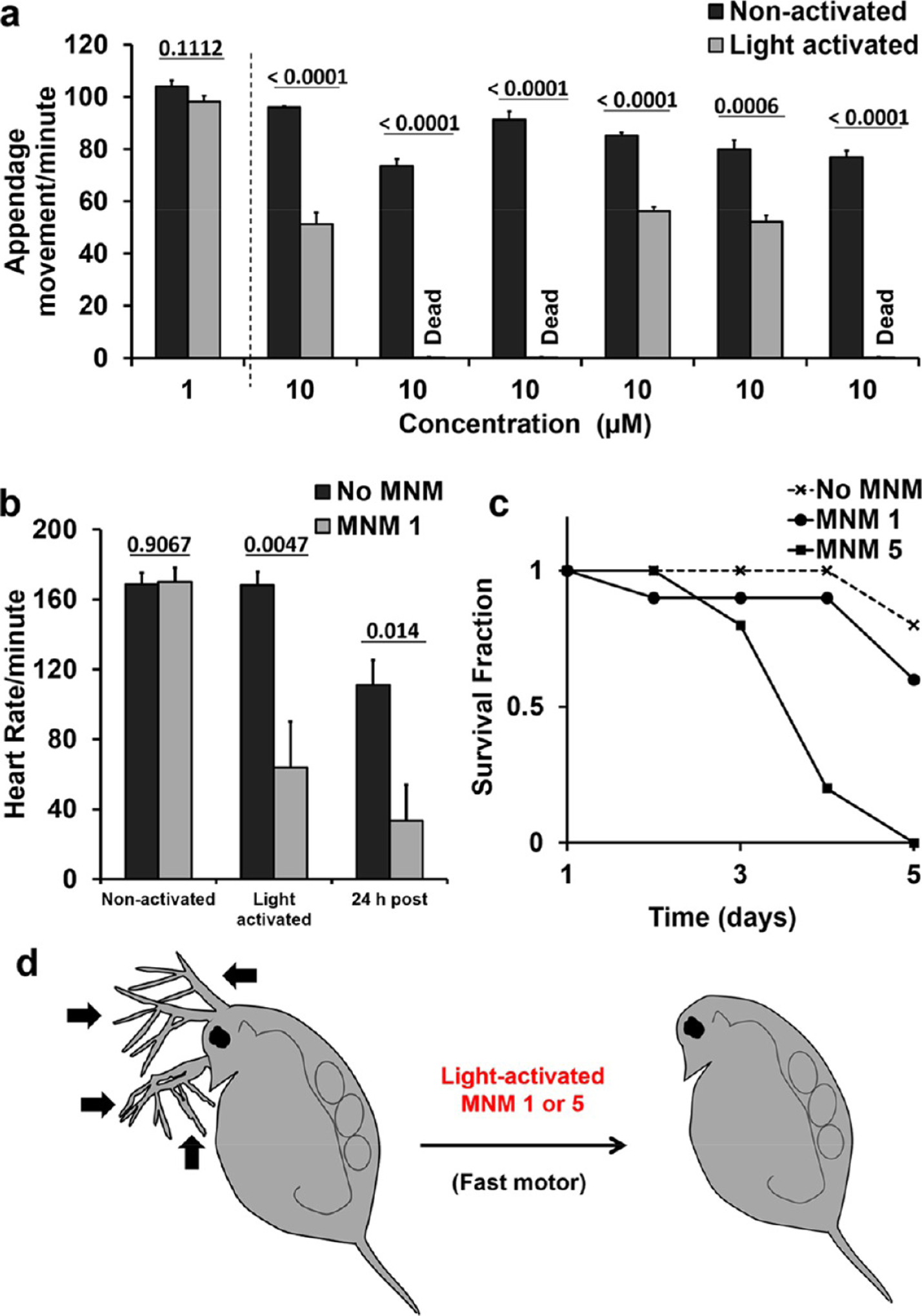
*Daphnia* exposed to light-activated MNM **1** shows physiological changes, and MNM **5** causes increased mortality. Adult *Daphnia* incubated with MNM for 10 min and subjected to 365 nm light activation for 10 min. (a) *Daphnia* exposed either 1 or 10 *μ*M MNM **1** with or without 365 nm light activation. Each bar represents one *Daphnia* to illustrate the changes in the rate of appendage movement before and after 365 nm light activation. (b) Five *Daphnia* exposed to no MNM (control) or 10 *μ*M MNM **1**, with or without light activation. Changes in heart rate observed in *Daphnia* exposed to either no MNM or MNM **1**. A significant decrease in heart rate is observed in the group exposed MNM **1** after 365 nm light activation (*P* = 0.0047) and 24 h postexposure (*P* = 0.014) compared to no MNM control. (c) Ten adult *Daphnia* exposed to 365 nm light-activated with no MNM or fast MNM **5** were followed for 5 days. This contained 1% DMSO. *Daphnia* exposed to MNM **5** showed higher mortality as compared to no MNM (*P* < 0.0001). (d) Schematic of a healthy adult *Daphnia* exposed to light-activated MNM **1** or **5** results in reduced heart rate, reduced limb movement, and limb dismemberment, eventually leading to its death. Black arrows indicate *Daphnia* appendages. Data points represent the mean of each group, and the error bars represent the standard error. *P*-values in (a, b) were generated by using unpaired two-tail *t* tests, and the horizontal bars indicate the two groups compared. The *P*-value in (c) was calculated with a log-rank Mantel–Cox test.

We evaluated the *in vivo* nanomechanical action of MNM **1** in 1% DMSO in *Daphnia pulex*. *Daphnia*, housed in a freshwater reservoir, were grown to adulthood and exposed to MNM **1**. *Daphnia*, in uniform water droplets, were incubated with 10 *μ*M MNM **1** for 10 min and then exposed to 10 min of 365 nm light. We examined whether the nanomechanical action of MNM **1** in *Daphnia* is dose-dependent by assaying 1 and 10 *μ*M concentrations. The appendage movement of each *Daphnia* was measured before and after 365 nm light activation of MNM **1**.^28^ Appendage movement in *Daphnia* exposed to 1 *μ*M MNM **1** did not change significantly (Figures 7a and 8a). In contrast, *Daphnia* exposed to 10 *μ*M MNM **1** showed reduced appendage movement in those individuals that remained alive and no movement in those that were dead (Figures 7b,c and 8a). The heart rate of *Daphnia* decreased from 170 to 60 beats min^−1^ after light activation of MNM **1** (Figure 8b). The heart rate remained close to 160 beats min^−1^ in the control group without MNMs. Control groups without MNMs were exposed to either 0.1% or 1% DMSO, based on the concentration of MNM used in the experimental groups. *Daphnia* were observed 24 h postexposure, and the heart rate of those exposed to light-activated MNM **1** continued to remain significantly lower than those not exposed to MNM **1** (Figure 8b). Representative individual *Daphnia* are shown to illustrate the changes observed, as some had appendages, but no movement after exposure (Figure 7a–c). These data suggest that light-activated MNM **1** can cause physiological changes and severe appendage tissue damage in *Daphnia*.

### Water-Soluble Light-Activated MNM 5 Causes Mortality in *Daphnia*.

Light-activated MNM **1** causes physiological changes and damage in *Daphnia*. We synthesized another MNM that is nearly identical to MNM **1** but carries a PEG arm to improve water solubility, and we examined its impact on *Daphnia*. Adult *Daphnia* were incubated with MNM **1** or MNM **5** for 10 min and activated with 365 nm light for 10 min. *Daphnia* incubated with light-activated MNM **5** showed higher mortality (100%) than without MNM by using the same light exposure (20%) 5 days postexposure (Figure 8c). MNM **5** displayed significantly higher mortality rates in *Daphnia* compared to MNM **1**. This is likely due to the higher solubility and bioavailability of MNM **5** compared to MNM **1** in aqueous solution. While the killing of *Daphnia* takes time, the physiological changes that occur due to fast rotating MNMs most likely led to their death (Figure 7c). We also observed appendage dismemberment in *Daphnia* exposed to light-activated MNM **5**, suggesting the damage caused by MNM **5** is very similar to that caused by MNM **1**. Our results show that *Daphnia* exposed to light-activated MNM **1** or **5** led to limb dismemberment, reduced heart rate, reduced limb movement, and eventually the death of *Daphnia* (Figure 8d). These observations demonstrate the ability of these light-activated fast motor MNMs to cause damage to cells and tissues in small eukaryotes.

### Light-Activated MNM 3 Can Cause Epidermal Ulcerations in Mouse Skin.

**Figure 9.**
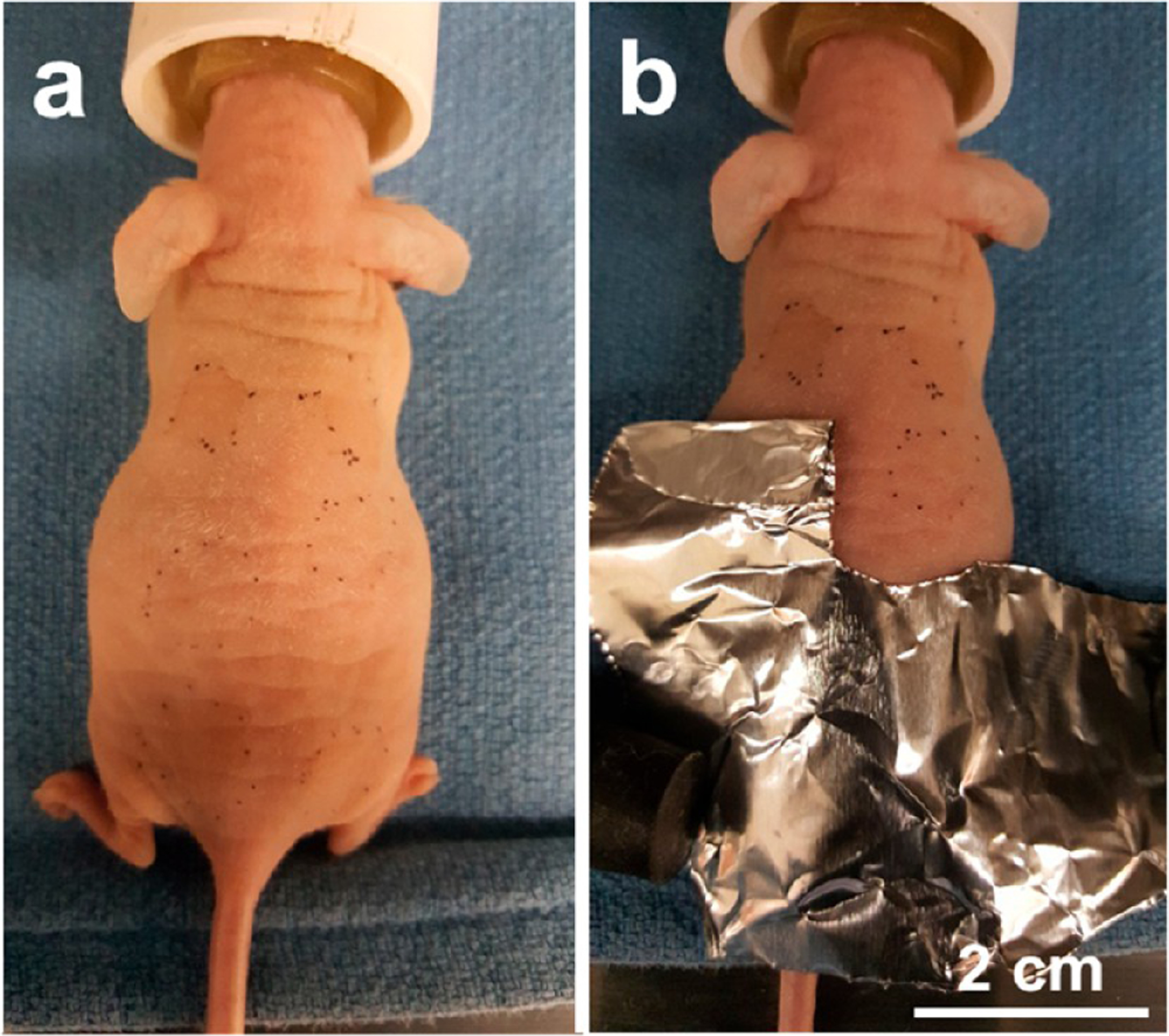
Anesthetized mouse treatment experimental setup. Swiss nu/nu nude mouse skin on the dorsal surface incubated with MNM and subjected to light activation. Six areas on the skin were incubated with MNM in acetonitrile or acetonitrile alone while under anesthesia. (a) Mouse before 365 nm light exposure. (b) Mouse skin area not exposed to light was covered with aluminum foil. Mice were exposed to 365 nm light at 20 mW cm^−2^ for 30 min.

**Figure 10.**
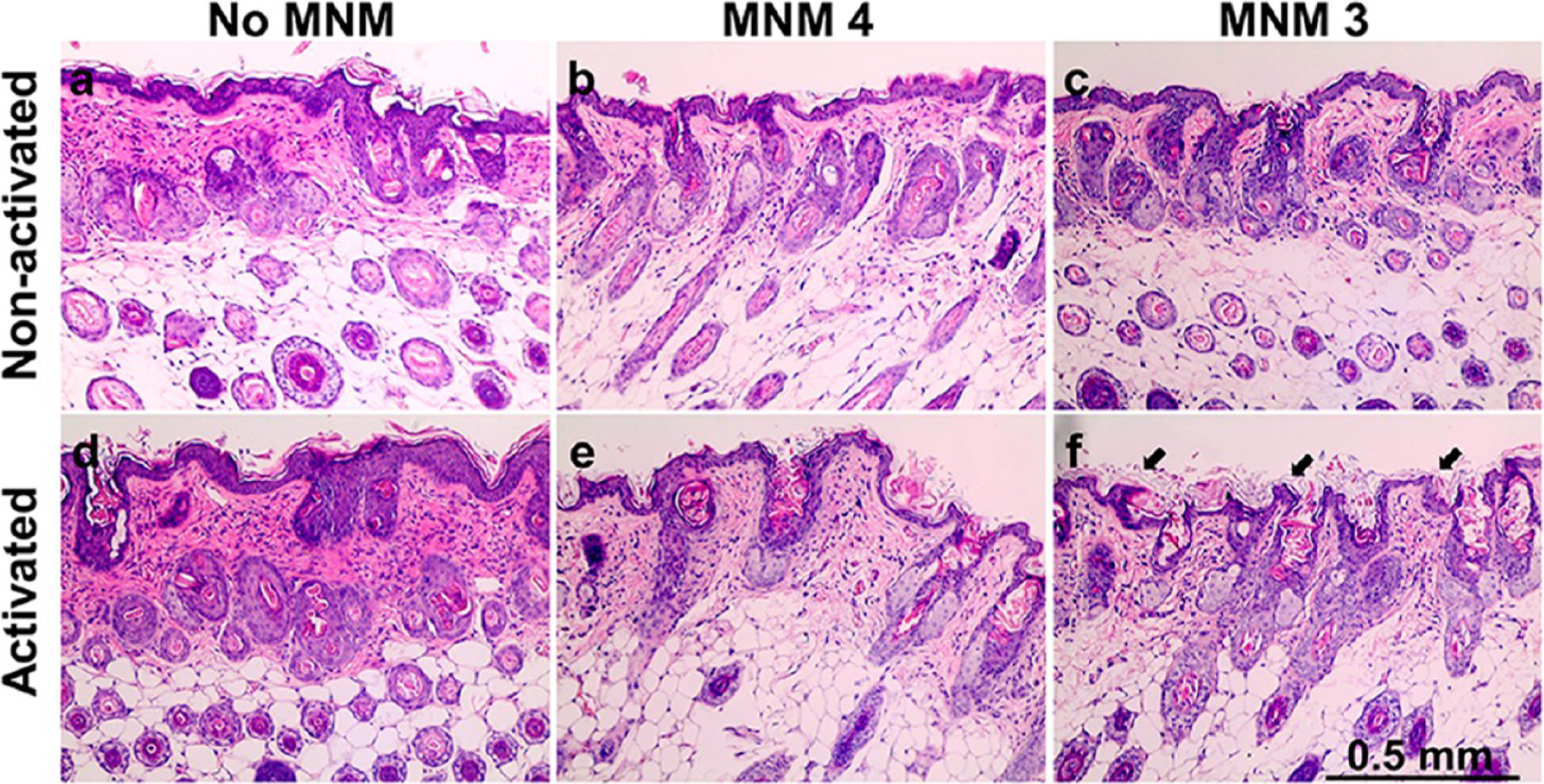
Mouse skin exposed to light-activated MNM **3** displays epidermal damage. Histopathology using 4 *μ*m thick mice skin sections, stained with hematoxylin and eosin (H&E). Sections were examined by using an optical microscope with a 20× objective. Swiss nu/nu nude mice exposed to 100 *μ*M MNM **3** or MNM **4**. The no MNM control was exposed to the solvent acetonitrile. (a–c) Sections of mouse skin that were not activated with 365 nm light. (a) Skin exposed to acetonitrile without MNM. (b) Skin exposed to MNM **4** dissolved in acetonitrile. (c) Skin exposed to MNM **3** dissolved in acetonitrile. (d–f) Sections of mouse skin that were activated with 365 nm light for 30 min. (d) Skin exposed to acetonitrile without MNM. (e) Skin exposed to light-activated MNM **4** dissolved in acetonitrile. (f) Skin exposed to light-activated MNM **3** dissolved in acetonitrile. Black arrows indicate epidermal ulceration and microlesions. The scale bar at the lower right is the same for all images.

We examined the activity of light-activated fast motor MNM on larger vertebrate animals. We used the BODIPY-labeled MNM **3** and MNM **4** to treat hairless nude mouse skin (Figure 9) so that we could both evaluate the effects of the rotation speed of MNM and track the localization of the MNM in tissues. Skin sections exposed to MNM **3** or MNM **4**, with or without light activation, were stained with hematoxylin and eosin for pathological examination (Figure 10). Acetonitrile was used to apply the MNM, but it evaporated before the light exposure. Acetonitrile is a common solvent used for skin treatment purposes.^29,30^ Histology sections in all three groups without light activation show intact epidermis (top purple layer) with preserved architecture and homogeneous thickness (Figure 10a–c). Similarly, mouse skin exposed light-activated slow MNM **4** or no MNM groups also had intact epidermis (Figure 10d,e). In contrast, the epidermis of skin exposed to light-activated fast MNM **3** displays widespread epidermal lesions and ulcerations (black arrows) (Figure 10f). The epidermis appeared thin, and the epidermal architecture was not fully intact in this exposure group. The skin segment exposed to light-activated MNM **4** shows a slight thinning of the epidermal layer but does not show ulcerations as observed with light-activated MNM **3** (Figure 10e). The control mouse skin exposed to 365 nm light with no MNM shows histological features similar to other controls, without any observable damage (Figure 10d). Overall, the histological examination suggests that light-activated MNM **3** can cause ulcerations and micro-lesions in the epidermis of mouse skin.

### Localization of MNM 3 in Mouse Skin upon Light Activation.

**Figure 11.**
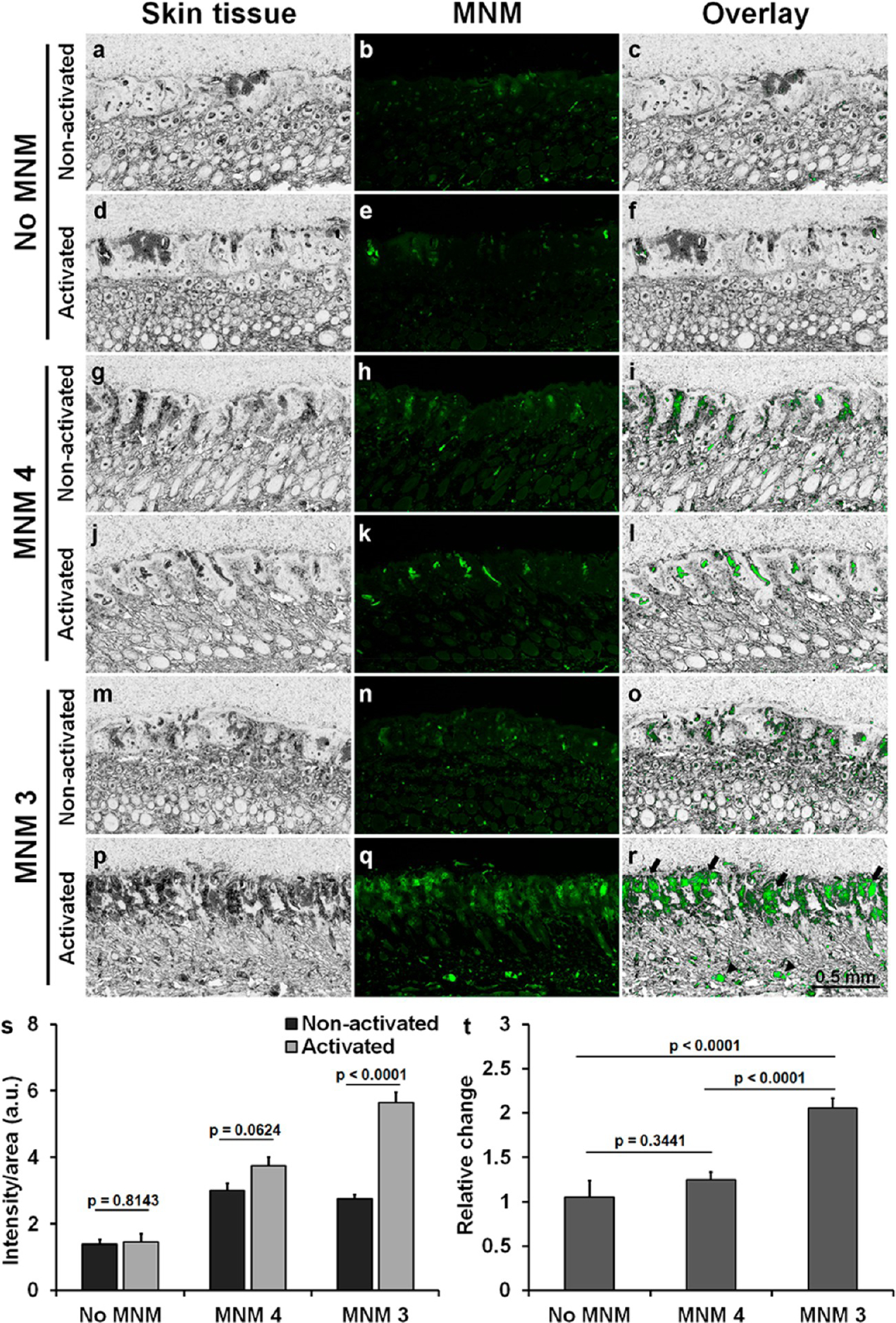
Light-activated fluorescent MNM **3** can localize to mouse skin epidermal and dermal layers. (a–r) Unstained 4 *μ*m thick mouse skin sections imaged using a Cytation 5 cell imaging multimode reader with a 4× 0.13 NA air objective using bright-field for skin tissue histology (a, d, g, j, m, and p), 469 nm excitation wavelength for MNM (b, e, h, k, n, and q), and overlay (c, f, i, l, o, and r). (a–f) Mouse skin exposed to acetonitrile without MNM: (a–c) no 365 nm light-activation, (d–f) activation with 365 nm light for 30 min. (g–l) Section of mouse skin exposed to MNM **4** dissolved in acetonitrile: (g–i) no 365 nm light-activation, (j–l) activation with 365 nm light for 30 min. (m–r) Mouse skin exposed to MNM **3** dissolved in acetonitrile. (m–o) no 365 nm light-activation, (p–r) activation with 365 nm light for 30 min. Black arrows indicate areas of MNM **3** localization to the epidermal layer. Black arrowheads show areas of MNM **3** localization in the dermal region. (s–t) Fluorescence of MNM localized to the epidermal layer. (s) Comparison of MNM fluorescence with and without light-activation in the no MNM, MNM **4**, and MNM **3** groups. (t) Relative change in MNM fluorescence with light activation compared among the three exposure groups; no MNM, MNM **4**, and MNM **3**. The data points represent the mean of each group using 16 epidermal sections, and error bars represent the standard error (SEM). *P*-values were obtained by using unpaired two-tail *t* tests, and the horizontal bars indicate the two groups compared. The scale bar at the lower right is the same for all images.

We examined mouse skin treated with MNM **3** and MNM **4** under fluorescent microscopy (Figure 11) to localize and quantify the MNM within the skin. Skin treated with nonactivated MNM **3** and MNM **4** display only background fluorescence (Figure 11a–c,g–i,m–o). Light-activated MNM **4** also displayed baseline levels of fluorescence (Figure 11d–f,j–l). These observations suggest that there are only low levels of MNM **3** or MNM **4** present in the epidermal layer of skin in the absence of activation. Also, activation of MNM **4** does not result in increased localization of the MNM. In contrast, we observed accumulation and localization of MNM **3** (black arrows) in the epidermal layer of the light-activated MNM **3** skin (Figure 11p–r). Compared to the light-activated MNM **4** or no MNM, light-activated MNM **3** skin displayed significantly higher BODIPY fluorescence (Figure 11s–t). These results suggest that light-activated MNM **3** can localize to the mouse epidermis and appear to penetrate deeper into the skin than in the absence of light activation or as compared to slow-rotating MNM **4**.

These observations suggest that the nanomechanical action of light-activated MNM **3** can be used to penetrate and infiltrate mouse skin. The apparent micro-lesions and ulcerations in mouse skin suggest that light-activated MNM might have localized therapeutic use for skin growth (cosmetic) or early carcinomas or melanomas treatment, not only in the superficial layer but also in the epidermis. Its localized application and activation allow a controlled environment for the destruction of several layers of skin or epithelial cells.

### Model for the Nanomechanical Action of MNM in *C. elegans*, *Daphnia*, and Mouse Skin.

**Figure 12.**
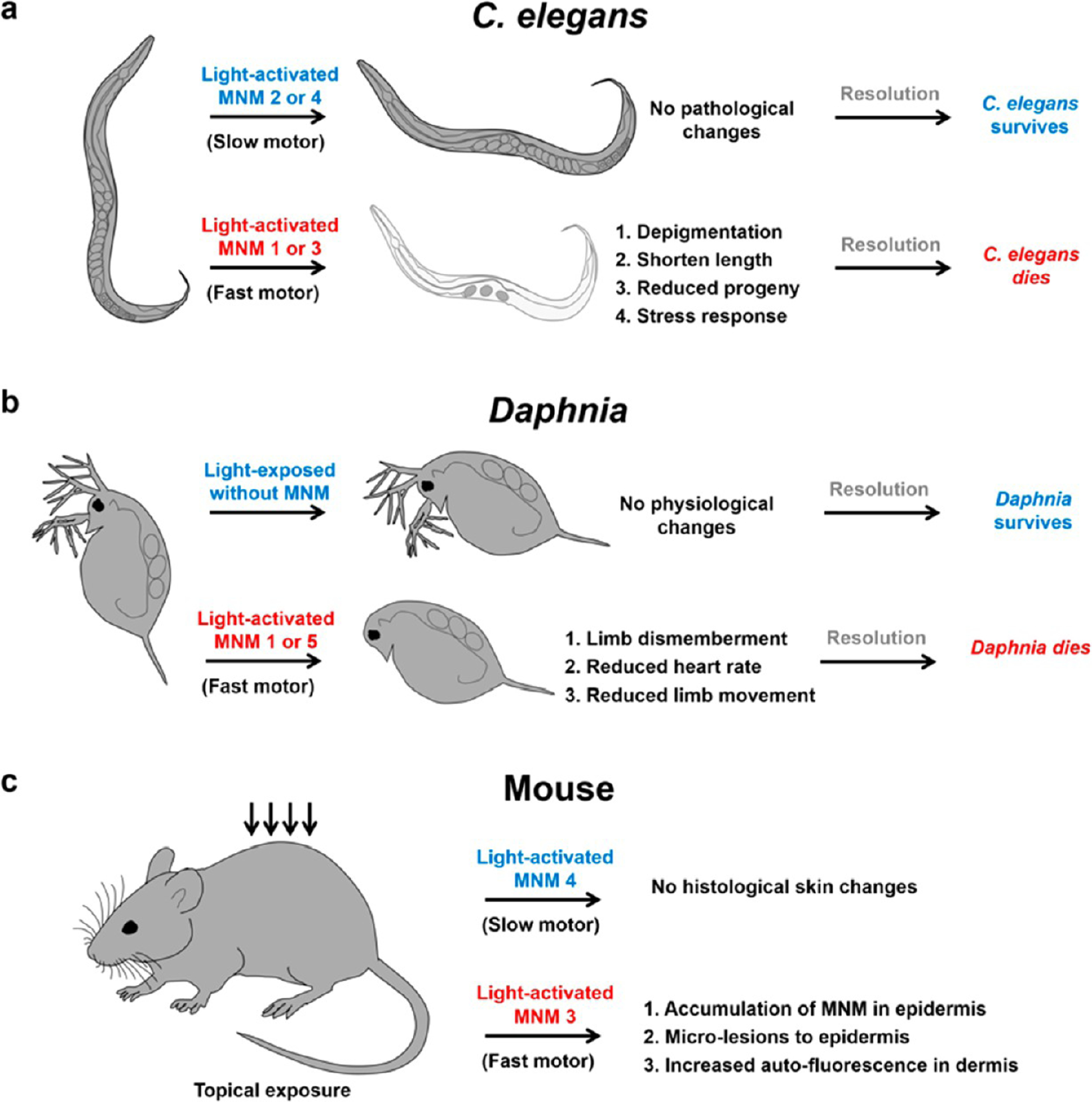
Model summarizing results from the nanomechanical action of MNM in *C. elegans*, *Daphnia pulex*, and mouse skin. MNM causes damage to cells and tissues in eukaryotic species by nanomechanical drilling action of the fast motor. (a) *C. elegans* exposed to light-activated MNM **1** or **3** caused depigmentation, increased the fragility of the cuticle, increased autofluorescence, and increased mortality of the nematode by 2 days postexposure. (b) *Daphnia* exposed to light-activated MNM **1** or **5** caused a decrease in heart rate, decreased appendage movement, dismemberment, and an increase in mortality by day five postexposure. (c) Mouse skin exposed to light-activated MNM **1** or **3** caused epidermal damage, displayed accumulation of MNM in the epidermis, and increased autofluorescence in the dermis.

*C. elegans* exposure to light-activated fast MNM **1** or **3** leads to depigmentation, shortening of their length, increasing of autofluorescence, and increasing mortality of the nematode by 2 days postexposure (Figure 12a). *Daphnia* exposed to light-activated fast MNM **1** or **5** causes a decrease in heart rate, decreased appendage movement, dismemberment of limbs, and an increase in mortality by 5 days postexposure (Figure 12b). Mouse skin exposed to light-activated fast MNM **3** displays epidermal ulcerations and micro-lesions in the skin, accumulation of MNM **3** in the epidermis, and increased autofluorescence in the dermal layer (Figure 12c). Our results show that the three fast MNM **1**, **3**, and **5** cause damage to cells and tissues in eukaryotic species most likely due to the nanomechanical drilling action of the fast motor.

This study extends the previous work showing *in vitro* activity of MNM **1** and **3** in destroying prostate cancer cells and mouse embryonic fibroblast cell lines.^21^ MNMs accelerated the death of these cells compared to the baseline necrosis observed by the effects of 365 nm light. In contrast, high mortality in eukaryotic species can be attributed to the fast drilling action of the three fast MNM **1**, **3**, and **5** while the control samples display much lower mortality during the same time. This was seen in both *C. elegans* and *Daphnia*, where *C. elegans* first showed depigmentation and *Daphnia* showed physiological changes such as slowing heart rate and appendage movement and finally limb dismemberment and death. This shows that light-activated fast motor MNM can extend its destructive properties to cells present in multicellular eukaryotes *in vivo* as well.

Furthermore, we recently showed the action of light-activated MNM **1** in prokaryotic species, helping to increases the susceptibility of an extensively drug-resistant *Klebsiella pneumoniae* to otherwise ineffective antibiotics.^22^ These studies, taken together, show the versatility of these light-activated MNMs and showcase their ability to disrupt the integrity of prokaryotic cell walls, eukaryotic cell membranes, and physiological functions of multicellular eukaryotes. While previous studies looked into applications of unidirectional molecular motors on surfaces of metal, in liquid crystalline environment and bilayers of lipids membranes, their applications in whole organisms have not been systematically studied.^2,31,32^ Our findings suggest potential broader applications of MNMs in biological and physiological systems warranting further systemic studies into their applications.

One potential limitation of the study is the use of 365 nm wavelength light to activate the MNM. This is in the UV-A spectrum and can sometimes have harmful effects on mammalian tissue over long exposure times. These MNM have been shown to be activated by two-photon excitation, which may be less likely to cause detrimental effects in mammalian cells.^33^ More recently, we synthesized MNM that can be activated by visible light (395–425 nm) that will expand the utility of MNM with increased safety. Moreover, that study further demonstrated that the effects of the MNM were not due to increased generation of reactive oxygen species (ROS). In fact, the twisted alkene in the MNM are free radical traps.^34^

## CONCLUSIONS

In summary, our study shows that the nanomechanical action of light-activated fast-rotating MNM (**1**, **3**, and **5**) causes varying degrees of damage to cells and tissues of eukaryotic species: *C. elegans*, *Daphnia*, and mice. These MNM also increase mortality in *C. elegans* (70%) and *Daphnia* (100%) over a few days postexposure. The depigmentation of *C. elegans* and the dismemberment of *Daphnia* limbs suggest that the action of MNM first causes nanomechanical disruption of cells and tissues and then causes death in organisms that cannot recover from MNM damage. Decreased heart rate and appendage movement in *Daphnia* also highlight the impact of light-activated fast motor MNM on physiology in small eukaryotes. Lesions and ulcerations caused by topical application of light-activated MNM onto mouse skin demonstrate the ability of MNM to function in larger eukaryotes. Light-activated MNMs have potent *in vivo* activity against microscopic eukaryotes and skin tissue, suggesting the potential to be applied to industrial or environmental parasite control, local treatment of diseases including skin cancer, and cosmetic application on the skin.

## MATERIALS AND METHODS

### Light Source to Activate MNMs.

A 365 nm light source (Sunlite Science & Technology, Inc., www.powerledlighting.com) was used to activate MNMs. MNMs were activated with a constant light flux by placing the light source directly above at a constant distance to the eukaryotic organism (Figure 2a–d). The 365 nm light source had a narrow wavelength spectrum of 360–376 nm, with a peak intensity at 368 nm and a constant power output of 10–15 mW/cm^2^.^22^

### Synthesis of the BODIPY Slow Motor 4.

Syntheses of MNM **1**, **2**, and **3** were described previously.^21,35^ A 2 mL vial charged with motor **2** (13.0 mg, 0.022 mmol), BODIPY dye^36^ (20.0 mg, 0.049 mmol), CuSO_4_·5H_2_O(s) (0.55 mg, 0.022 mmol), and sodium ascorbate (0.93 mg, 0.007 mmol) was sealed with a rubber septum cap. A well-degassed mixture of CH_2_Cl_2_ (0.1 mL) and water (0.1 mL) was added to the vial, and the vial was shaken by a wrist-action shaking machine for 36 h. The mixture was partitioned between CH_2_Cl_2_ (5 mL) and water (5 mL). The organic phase was dried over anhydrous MgSO_4_ and filtered, and the filtrate was concentrated under vacuum. The crude product was purified by preparative TLC (silica gel, 4% MeOH in CH_2_Cl_2_) to afford the desired compound **4** as an orange solid (27 mg, 87%). Complete spectra are provided in the Supporting Information (Figures S2–S4).

### *C. elegans*, *Daphnia*, and Mice Strains.

N2 (Bristol, wild type) and TP12 [*kaIs12*(col-19::GFP)] *C. elegans* strains were used in this study. The N2 strain was used to observe pathological changes and for mortality assays. The TP12 strain was used for confocal microscopy imaging with fluorescent MNM-BODIPY in *C. elegans*. The *Daphnia* strain was obtained from Carolina Biological Supply Company (Burlington, NC). Female Swiss nu/nu nude mice were maintained and handled using protocols approved by the Institutional Care and Use Committee (IACUC) at the MD Anderson Cancer Center (MDACC).

### *C. elegans* Exposure to MNM.

Adult *C. elegans* (N2) from a nonstarved NGM plate was subjected to a bleaching procedure to obtain nematode eggs.^37^ Age synchronized 3-day-old adult N2 *C. elegans* nematodes recovered with M9 buffer were used, and about 200 worms each were placed Eppendorf tubes in 1 mL of M9 buffer. *C. elegans* were exposed to MNM **1**, MNM **2**, or a DMSO control without MNM at concentrations of 1, 10, or 100 *μ*M. Nematodes were incubated at room temperature for 15 min with gentle shaking on a test tube rocker. Worms were pipetted onto small NGM plates seeded with OP50 and exposed to 365 nm light for 15 min or not exposed to light. The worms were then placed at room temperature (20–22 °C) overnight and followed for the duration of the worms’ life spans. Worms were counted daily and transferred onto new small NGM agar plates for the first 3 days after light exposure and then transferred onto new plates every other day.

### Imaging of *C. elegans*.

TP12 [*kaIs12*(col-19::GFP)] *C. elegans* expressing cuticular eGFP under the control of gene *col-19* were exposed to MNM **3** or **4** as detailed above. After exposure, *C. elegans* were fixed with 4% paraformaldehyde and washed once with M9 buffer. *C. elegans* were then mounted onto slides, and 4–6 worms were imaged in each exposure group. Each nematode was imaged at the head (upper), trunk (middle), and tail (lower) region, at a magnification of 400× and a resolution of 1024. Representative images are presented to show differences observed after exposure to nonactivated and activated MNM. A confocal microscope (Nikon A1R+/A1+) with a FITC fluorescence filter (excitation 488 nm, emission 525/50 nm) and 40× oil immersion objective were used. Confocal microscopy used the Galvanometric scanning method controlled by a 1/4 frame/s (fps: 0.25; frame time 4 s), immersion oil type F (*η* = 1.518), a numerical aperture of 1.3, and a pinhole of 0.6 AU (19.2 *μ*m). The size of the images was 1024 × 1024 pixels, mono 12 bit (0.31 *μ*m/px), and an average of two images were taken.

### Confocal Microscopy Quantification.

NIH ImageJ (version 1.4.3.67) software was used to quantify fluorescent intensities of images obtained by confocal imaging. Confocal images were obtained with a FITC fluorescence filter (excitation 488 nm, emission 525 nm) and 40× 1.3 NA oil immersion objective. For quantification of fluorescence, each group containing four nematodes with three different segments (head, trunk, and tail) were used. The fluorescence signal in three regions within each segment was quantified by using the same area, and averages were calculated. For each group, the ratios of non-light-activated to light-activated MNM were obtained, and their averages were calculated. Results presented are an average of four images taken for each nematode segment in each group. The total sample size was at least four *C. elegans* per exposure group, with images of the upper, middle, and lower sections of each worm. When more than four *C. elegans* were imaged, it is stated in the results. For quantification, the worm images were further divided into three areas to give a total of 12 representative areas for each *C. elegans* segment.

### *Daphnia* Exposure to MNM.

Adult *Daphnia* were incubated with MNM **1** for 10 min and exposed to 365 nm light for 10 min, similar to the setup described for *C. elegans*. Heart rate and appendage movement were measured on the same *Daphnia*, before and after light activation of MNM **1**. Observations were made under 20× magnification with a light microscope for 15 or 30 s, and the rates were calculated per minute. Ten adult *Daphnia* were incubated with MNM **5** for survival assay. The *Daphnia* exposed either to no MNM or MNM **5** were followed over the course of 5 days for mortality and survival.

### Mouse Skin Exposure to MNM.

Female Swiss nu/nu nude mice were obtained from the Experimental Radiation Oncology Mouse Facility at the MD Anderson Cancer Center. They were treated with 10 *μ*L of 100 *μ*M MNM **3**, MNM **4** or no MNM in acetonitrile, over six delimited areas on their skin while anesthetized with isoflurane administered. After 5 min of incubation for the acetonitrile solvent to allow it to evaporate, half the mouse skin that had been topically treated with MNM was exposed to 365 nm light at 20 mW/cm^2^ for 30 min and the other half was covered with aluminum foil to protect it from light exposure (Figure 9). The six exposure areas were as follows: (1) MNM **3** (fast motor-BODIPY) without light activation; (2) MNM **4** (slow motor-BODIPY) without light activation; (3) no MNM control (acetonitrile) without light exposure; (4) MNM **3** (fast motor-BODIPY) with light activation; (5) MNM **4** (slow motor-BODIPY) with light activation; (6) no MNM control (acetonitrile) with light exposure. Immediately after the light activation, the mice were euthanized and skin segments were harvested. MD Anderson institutional animal care and use committee (IACUC) protocols were followed when handling the mice.

### Histopathology of Mouse Skin Exposed to MNM.

Mouse skin segments were fixed in 10% buffered formalin for 24 h and embedded in paraffin blocks. The skin tissues were processed as 4 *μ*m thick sections and subsequently stained with hematoxylin and eosin (H&E). H&E stained slides were imaged using an optical microscope (AMEX-1100, Advance Microscopy Group) with a 20× objective.

### Fluorescent Microscopy of Mouse Skin Exposed to MNM.

Mouse skin segments were fixed in 10% buffered formalin for 24 h and embedded in paraffin blocks prior to cutting 4 *μ*m thick sections. These unstained slides were imaged for NM-BODIPY emission by using an inverted fluorescence microscope (DMI6000B, Leica, Germany) with a 20× objective, 0.4, L5 blue light filter cube with excitation filter 480/40 nm and suppression filter 527/30 nm. The tissue autofluorescence was imaged with N3 green light filter cube with excitation filter 546/12 nm and suppression filter 600/40 nm. The fluorescence intensities were quantified by using ImageJ with a similar method as used in *C. elegans*. Fluorescence images for localization were prepared on a Cytation 5 cell imaging multimode reader with a 4× objective using bright-field for skin tissue histology and 469 nm excitation wavelength for BODIPY-MNM.

### Statistical Analyses.

The sample size and numbers of replicates used are stated in each Figure legend. GraphPad was used to perform statistical analyses of different exposure groups. Means and standard errors presented in each Figure were plotted in Microsoft Excel.

## Supplementary Material

SupportingInformation
