# Supplementary material for: Molecular Nanomachines Can Destroy Tissue or Kill Multicellular Eukaryotes
Source: ACS Appl Mater Interfaces. Author manuscript; Available in PMC 2021 Jun 9. (PMC8189693; doi:10.1021/acsami.9b22595)
Supplement: SupportingInformation [file NIHMS1708387-supplement-SupportingInformation.pdf]

Supporting Information

Molecular Nanomachines Can Destroy Tissue or Kill Multicellular Eukaryotes

Richard S. Gunasekera,[†,⊥,‡,] Thushara Galbadage,[ʃ,‡] Ciceron Ayala-Orozco,[†,∥] Dongdong Liu,[†]*

Victor García-López,[†] Brian E. Troutman,[¶] Josiah J. Tour,[ʃ] Robert Pal,[ξ] Sunil Krishnan,[∥,1]

*Jeffrey D. Cirillo,[ʃ] and James M. Tour[†,¥,]**

[†]Department of Chemistry, [¶]Department of Physics and Astronomy, [¥]Department of Materials Science and NanoEngineering, [¥]Smalley-Curl Institute and [¥]NanoCarbon Center, Rice University, Houston, Texas 77005, United States.

[⊥]Department Biological Science, [§]Department of Chemistry, Physics and Engineering, Biola University, La Mirada, California 90639, United States.

[ʃ] Department of Microbial Pathogenesis and Immunology, Texas A&M Health Science Center, Bryan, Texas 77807, United States.

[∥]Department of Experimental Oncology, MD Anderson Cancer Center, Houston, Texas 77030, United States.

[ξ]Department of Chemistry, Durham University, South Road, DH1 3LE Durham, United Kingdom

‡These authors contributed equally

*Corresponding authors

E-mail: richard.gunasekera@biola.edu, tour@rice.edu

[1] Present address: Mayo Clinic Florida, Department of Radiation Oncology, 4500 San Pablo Blvd. S, Jacksonville, FL 32224.

Supporting Synthetic Methods

Synthesis of MNM 4.

The syntheses of MNM **1**, **2**, and **3** were reported previously.[1,2] MNM **4** was synthetized using cycloaddition between MNM **6** and the corresponding alkyne **7** (Sup Figure 1).

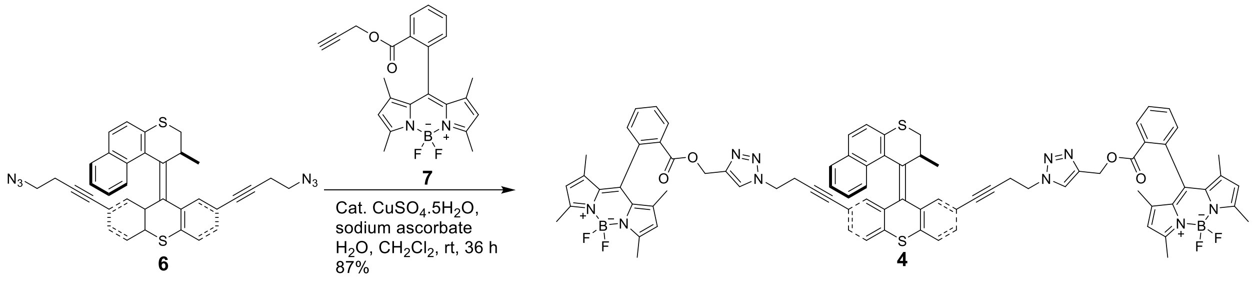

Figure S1. Synthesis of MNM 4. A 2 mL vial charged with MNM **6**[1] (13.0 mg, 0.022 mmol), BODIPY dye **7**[3] (20.0 mg, 0.049 mmol), $CuSO_4.5H_2O$(s) (0.55 mg, 0.022 mmol) and sodium ascorbate (0.93 mg, 0.007 mmol) was sealed with a rubber septum cap. A well degassed mixture of CH_2Cl_2 (0.1 mL) and water (0.1 mL) was added to the vial, and the vial was shaken by a wrist-action shaking machine for 36 h. The mixture was partitioned between CH_2Cl_2 (5 mL) and water (5 mL). The organic phase was dried over anhydrous $MgSO_4$, filtered, and the filtrate was concentrated under vacuum. The crude product was purified by preparative TLC (silica gel, 4% MeOH in CH_2Cl_2) to afford the desired compound **4** as an orange solid (27 mg, 87%): ^{1}H NMR (600 MHz, $CDCl_3$) δ 8.10 (ddd, *J* = 10.8, 7.9, 1.4 Hz, 2H), 7.68 – 7.60 (m, 3H), 7.59 – 7.54 (m, 4H), 7.50 (d, *J* = 8.1 Hz, 1H), 7.46 (d, *J* = 8.4 Hz, 1H), 7.39 (d, *J* = 8.5 Hz, 1H), 7.35 (s, 1H), 7.29 (ddd, *J* = 7.7, 4.7, 1.4 Hz, 2H), 7.27 (m, 1H, overlap with $CDCl_3$), 7.16 (s, 1H), 7.14 (d, *J* = 8.0 Hz, 1H), 7.10 (ddd, *J* = 8.1, 6.8, 1.2 Hz, 1H), 6.99 (ddd, *J* = 8.3, 6.8, 1.3 Hz, 1H), 6.67 (dd, *J* = 8.1, 1.8 Hz, 1H), 6.31 (d, *J* = 1.8 Hz, 1H), 5.93 (s, 1H), 5.92 (s, 1H), 5.91 (s, 1H), 5.85 (s, 1H),

5.29 (s, 2H), 5.26 (s, 2H), 4.55 (t, J = 7.2 Hz, 2H), 4.33 (td, J = 7.4, 2.4 Hz, 2H), 4.07 (m, 1H), 3.71 (dd, J = 11.4, 7.4 Hz, 1H), 3.08 (dd, J = 11.4, 3.4 Hz, 1H), 3.01 (t, J = 7.2 Hz, 2H), 2.71 (t, J = 7.3 Hz, 2H), 2.57 (s, 6H), 2.53 (s, 6H), 1.29 (s, 6H), 1.27 (s, 3H), 1.26 (s, 3H), 0.80 (d, J = 6.7 Hz, 3H). ^{13}C NMR (150 MHz, $CDCl_3$) δ 210.84, 206.96, 166.13, 166.07, 155.02, 154.99, 154.93, 142.62, 142.56, 142.47, 141.95, 141.58, 141.13, 141.10, 138.17, 137.85, 136.17, 135.68, 135.56, 135.32, 135.22, 134.25, 133.09, 132.14, 132.07, 131.58, 131.25, 131.21, 130.83, 130.78, 130.64, 130.58, 130.20, 130.15, 130.03, 129.56, 129.52, 129.46, 129.22, 129.17, 127.74, 127.66, 126.23, 125.97, 125.60, 124.49, 123.92, 123.81, 121.23, 121.20, 121.06, 120.11, 85.71, 84.31, 82.85, 82.27, 77.23, 77.02, 76.81, 59.24, 59.20, 48.77, 48.71, 37.13, 32.70, 32.62, 21.63, 21.42, 19.24, 14.69, 14.62, 14.05, 14.01. HRMS (ESI) m/z calculated for $[M+Na]^+$ $C_{81}H_{68}N_{10}O_4B_2F_4S_2$ 1429.4904, found 1429.4919.

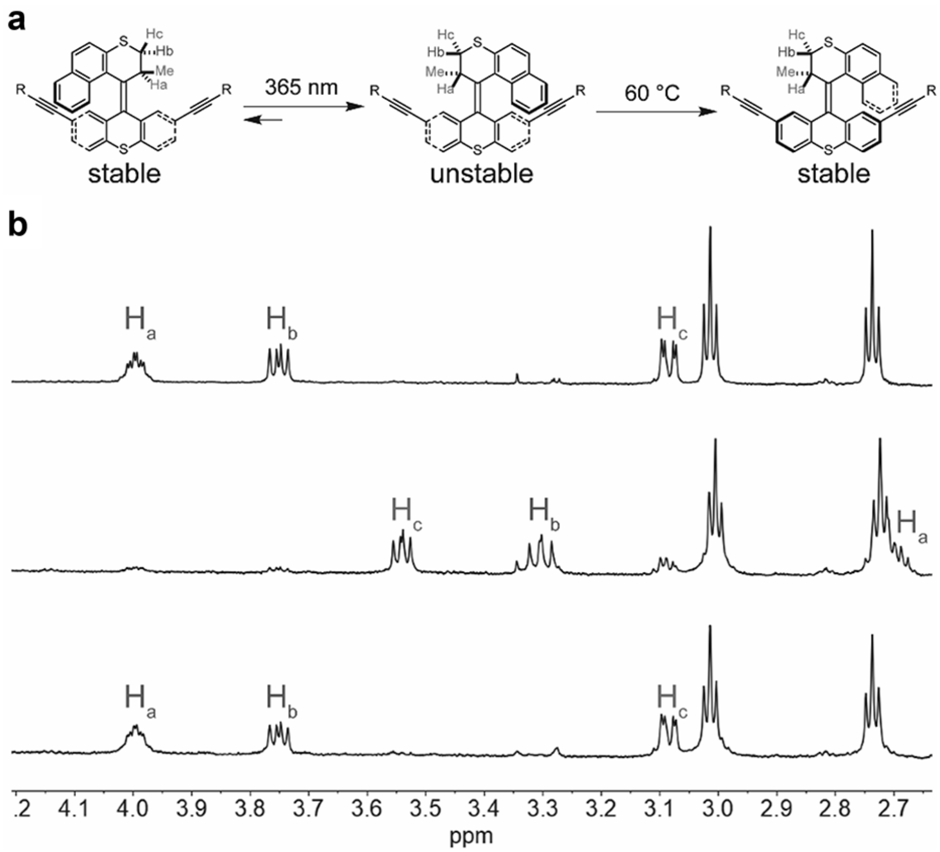

Figure S2. Partial ^{1}H NMR spectra of half-rotation of the slow motor **4** (1 mM in CD_3CN). (a) Schematic representation of half rotation of the slow motor **4**. (b) Partial ^{1}H NMR spectra of slow motor **4** before light activation (top), after light activation at 365 nm for 1 h showing 88% photoisomerization conversion (middle), and thermal helix inversion after heating the sample at 60 °C for 1 h forming the stable isomer in 99% (bottom). The motor without BODIPY shows similar conversion upon irradiation and heat.[24] Therefore, the BODIPY moieties do not impede the rotary action of the MNMs.

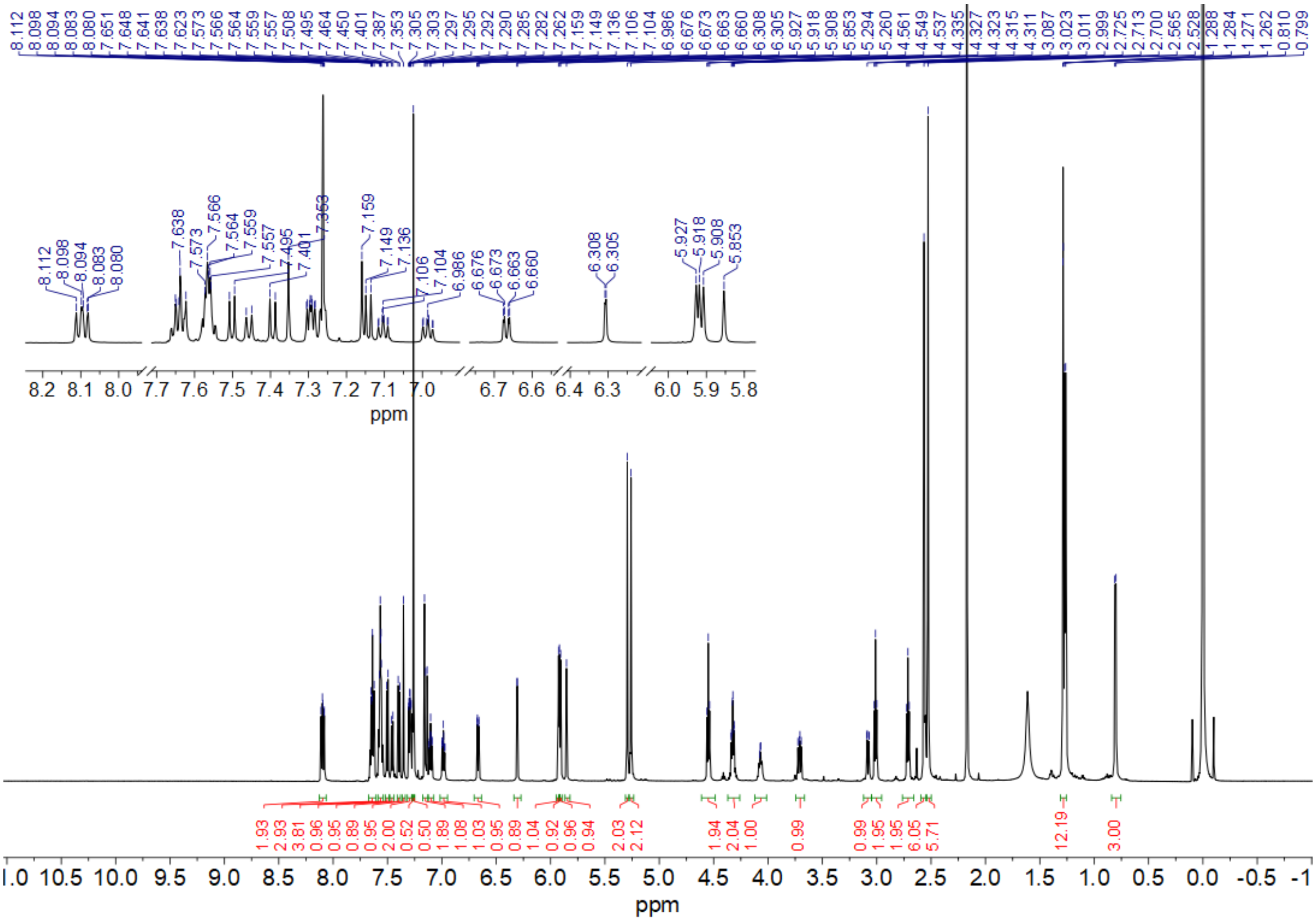

Figure S3. ^{1}H NMR spectrum (600 MHz, T = 298 K) of **4** in $CDCl_3$.

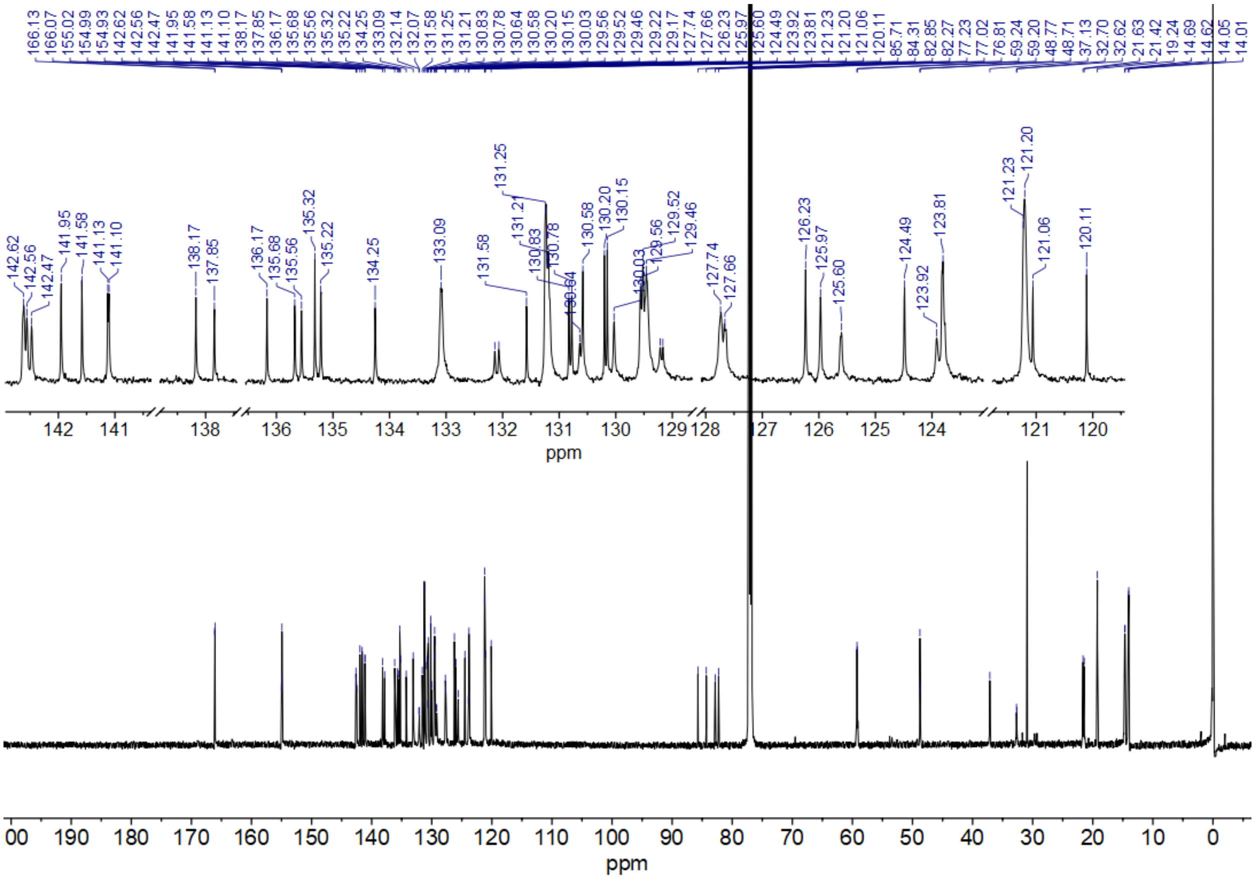

Figure S4. ^{13}C NMR spectrum (150 MHz, T = 298 K) of **4** in $CDCl_3$.

Supporting References

1. García-López, V.; Chiang, P. T.; Chen, F.; Ruan, G.; Marti, A. A.; Kolomeisky, A. B.; Wang, G.; Tour, J. M. Unimolecular Submersible Nanomachines. Synthesis, Actuation, and Monitoring. *Nano Lett*. **2015,** *15*, 8229-8239.

2. García-López, V.; Chen, F.; Nilewski, L. G.; Duret, G.; Aliyan, A.; Kolomeisky, A. B.; Robinson, J. T.; Wang, G.; Pal, R.; Tour, J. M. Molecular Machines Open Cell Membranes. *Nature* **2017**, *548*, 567-572.

3. Jin, T.; García-López, V.; Chen, F.; Tour, J.; Wang, G., Imaging Single Molecular Machines Attached with Two BODIPY Dyes at the Air–Solid Interface: High Probability of Single-Step-Like Photobleaching and Nonscaling Intensity. *J. Phys. Chem*. C **2016**, *120*, 26522-26531.
